# Fine‐Tuning X‐Ray Sensitivity in Organic–Inorganic Hybrids via an Unprecedented Mixed‐Ligand Strategy

**DOI:** 10.1002/advs.202305378

**Published:** 2023-11-08

**Authors:** Huangjie Lu, Zhaofa Zheng, Huiliang Hou, Yaoyao Bai, Jie Qiu, Jian‐Qiang Wang, Jian Lin

**Affiliations:** ^1^ Shanghai Institute of Applied Physics Chinese Academy of Sciences 2019 Jia Luo Road Shanghai 201800 P. R. China; ^2^ School of Nuclear Science and Technology Xi'an Jiaotong University No.28, West Xianning Road Xi'an 710049 P. R. China

**Keywords:** fluorochromic nanoclusters, mixed‐ligand, photochromic responses, radiation detection, thorium

## Abstract

Crystalline organic–inorganic hybrids, which exhibit colorimetric responses to ionizing radiation, have recently been recognized as promising alternatives to conventional X‐ray dosimeters. However, X‐ray‐responsive organic–inorganic hybrids are scarce and the strategy to fine‐tune their detection sensitivity remains elusive. Herein, an unprecedented mixed‐ligand strategy is reported to modulate the X‐ray detection efficacy of organic–inorganic hybrids. Deliberately blending the stimuli‐responsive terpyridine carboxylate ligand (tpc^−^) and the auxiliary pba^−^ group with different ratios gives rise to two OD thorium‐bearing clusters (**Th‐102** and **Th‐103**) and a 1D coordination polymer (**Th‐104**). Notably, distinct X‐ray sensitivity is evident as a function of molar ratio of the tpc^−^ ligand, following the trend of **Th‐102** > **Th‐103** > **Th‐104**. Moreover, **Th‐102**, which is exclusively built from the tpc^−^ ligands with the highest degree of *π*–*π* interactions, exhibits the most sensitive radiochromic and fluorochromic responses toward X‐ray with the lowest detection limit of 1.5 mGy. The study anticipates that this mixed‐ligand strategy will be a versatile approach to tune the X‐ray sensing efficacy of organic–inorganic hybrids.

## Graphical abstract

An unprecedented mixed‐ligand strategy to modulate the X‐ray detection efficacy of organic–inorganic hybrids is developed by deliberately blending of 2,2′:6′,2′'‐terpyridine‐4′‐carboxylate (tpc^−^) and 3‐(pyridin‐4‐yl)benzoate (pba^−^) ligands with different molar ratios, giving rise to a sensitive X‐ray dosimeter **Th‐102** with the lowest detection limit of 1.5 mGy.

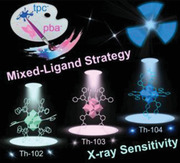

## 1 Introduction

The detection of X‐ray radiation is of great importance due to its indispensable role in scientifically, medically, and industrially relevant activities.^[^ ^1^ ^]^ Depending on the purpose, X‐ray doses ranging from mGy to Gy and up to kGy levels, have been employed for diagnostic, therapy, and sterilization, respectively.^[^ ^2^ ^]^ Therefore, dosimeters with satisfactory sensitivity, extending over orders of magnitude, are paramount to monitor the actual cumulative dose delivered to the targets. The sensitivity of X‐ray detectors can be tuned through a variety of strategies based on their detection mechanisms. For instance, integrating the advantages of high stopping power, low trap density, and high charge collection efficiency in semiconductor‐based detectors can enhance their sensitivity to X‐ray.^[^ ^3^ ^]^ Moreover, high light yields, large Stokes shifts, fast decay times, and high *Z*_eff_ constituents, as well as desirable emission wavelengths that match well with the photomultiplier, are important figures of merit for boosting the sensing efficiency of scintillators.^[^ ^4^ ^]^ Despite considerable progress in the development of highly sensitive semiconductors and scintillators for X‐ray detection, these X‐ray detectors are suitable for monitoring the dose rate rather than the accumulated dose of X‐ray, making them particularly applicable in the fields of X‐ray imaging and monitoring.^[^ ^5^ ^]^ Furthermore, the complex system configuration and high cost of semiconductors and scintillators further hinder their widespread applications in X‐ray dosimetry.^[^ ^5^ ^]^

Colorimetric dosimeters based on crystalline inorganic–organic hybrids are considered as promising alternatives due to their rich engineering toolbox for tailoring their structure and functionality at the molecular level.^[^ ^6^ ^]^ The spectroscopic changes and associated color transition upon continuous ionizing irradiation can be utilized for X‐ray dosimetry, providing the capability to inspect radiation dose in real‐time with a high degree of simplicity.^[^ ^7^ ^]^ This attribute can be exemplified by various radiochromic coordination polymers and a series of radio‐induced‐fluorochromic nanoclusters, whose instinct color and photoluminescence color can be correlated with the X‐ray dose, respectively.^[^ ^8^ ^]^ Despite these advancements, colorimetric X‐ray dosimeters remain scarce, particularly when compared with the semiconductor‐ and scintillator‐based detectors. Moreover, studies on the correlations between composition or structure and sensing efficacy are still in their infancy. The strategy to fine‐tune the detection sensitivity of colorimetric X‐ray dosimeters remains elusive, to the best of our knowledge.

One of the approaches for optimizing the properties of inorganic–organic hybrids, such as storage capacity, separation efficacy, catalytic activity, etc., is to introduce heterogeneity by incorporating multiple organic ligands.^[^ ^9^ ^]^ These early investigations encouraged us to explore the tunability of sensitivity by creating novel mixed‐ligand inorganic–organic hybrids. We speculated that tuning the stoichiometry of a radio‐responsive ligand and an auxiliary ligand in a single inorganic–organic hybrid material can not only lead to alternating structures but also sophisticate the intermolecular interaction of radio‐responsive ligand and ultimately modulate the detection sensitivity. In this work, we demonstrate a mixed‐ligand strategy for fine‐tuning the UV and X‐ray sensitivity of organic–inorganic hybrids. Deliberately blending of 2,2′:6′,2′'‐terpyridine‐4′‐carboxylate (tpc^−^) and 3‐(pyridin‐4‐yl)benzoate (pba^−^) ligands gives rise to three thorium‐bearing crystalline complexes, whose UV and X‐ray detection efficacy is correlated with the stoichiometry of the organic ligands. [Th_6_(OH)_4_(O)_4_(H_2_O)_6_(tpc)_11_(HCOO)]·8H_2_O (**Th‐102**), which is exclusively composed of tpc^−^ ligands with the highest degree of *π–π* interactions, exhibits the lowest detection limit of 1.5 mGy among the three materials.

## 2 Results and Discussion

### 2.1 The Assembly of Thorium Complexes Based on Different Ligand Stoichiometry

**Figure 1:**
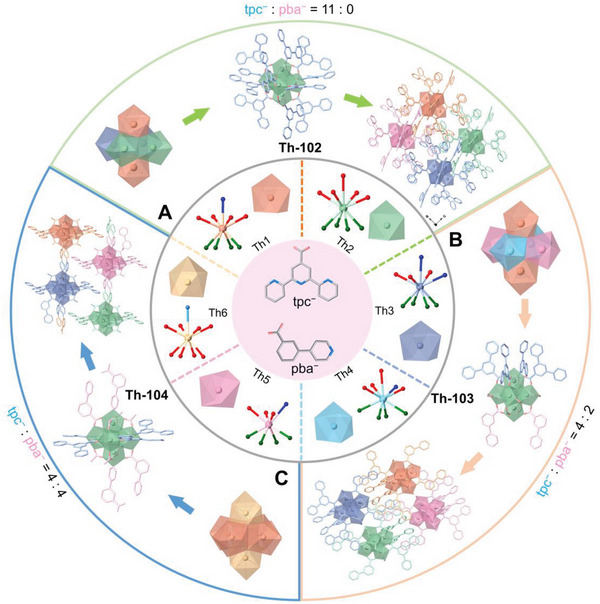
The assemblies of thorium‐based clusters and coordination polymer with different tpc^−^ and pba^−^ stoichiometry. a) Representation showing the inorganic SBU and the structure of Th‐102. b) Representation showing the inorganic SBU and the structure of Th‐103. c) Representation showing the inorganic SBU and the structure of Th‐104.

The solvothermal reaction between Th(NO_3_)_4_·6H_2_O, 2,2′:6′,2′'‐terpyridine‐4′‐carboxylic acid (Htpc), and perchloric acid in DMF/H_2_O solution affords a 0D thorium‐bearing cluster, **Th‐102**. Having successfully prepared **Th‐102** as a mono‐ligand benchmark material, we next sought to build mixed‐ligand complexes based on Htpc and 3‐(pyridin‐4‐yl)benzoic acid (Hpba). Comparable synthetic conditions with the addition of Hpba result in the crystallizations of another 0D cluster [Th_6_(OH)_4_(O)_4_(H_2_O)_6_(tpc)_4_(pba)_2_(HCOO)_6_]·2HCOOH·7H_2_O (**Th‐103**) and a 1D coordination polymer [Th_6_(OH)_4_(O)_4_(H_2_O)_4_(tpc)_4_(pba)_3.32_(CH_3_COO)_4.68_]·1.32DMF·H_2_O (**Th‐104**), both of which incorporate tpc^−^ and pba^−^ ligands but with different stoichiometry (**Figure** **1**).

Single crystal X‐ray diffraction (SCXRD) revealed that **Th‐102** crystalizes in a monoclinic space group *C*2/*c* (Table S1, Supporting Information). The [Th_6_(*µ*_3_‐OH)_4_(*µ*_3_‐O)_4_(H_2_O)_6_]^12+^ secondary building unit (SBU) of **Th‐102** is 12‐coordinated via carboxylate groups that are contributed from eleven tpc^−^ ligands and one HCOO^−^ anion in a *µ*_2_‐*η* ^1^ *,η* ^1^ terminal coordinating manner. Such a coordination manner gives rise to a 0D cluster structure of **Th‐102**. The afforded discrete Th_6_ clusters are packed into a crystalline lattice via the *π–π* interactions between adjacent tpc^−^ ligands (Figure 1a). **Th‐103** crystalizes in an orthorhombic space group *Pccn*. Each inorganic [Th_6_(*µ*_3_‐OH)_4_(*µ*_3_‐O)_4_(H_2_O)_6_]^12+^ SBU of **Th‐103** is twelve‐coordinated with four tpc^−^ ligands, two pba^−^ ligands, and six HCOO^−^ anions. The carboxylate groups of tpc^−^ and pba^−^ ligands involve in the coordination with the metal centers, while their terpyridine or pyridine moieties do not participate in bonding. Such coordination manners render tpc^−^ and pba^−^ as monotopic ligands, terminating the interconnection between the neighboring inorganic SBUs and affording 0D discrete clusters. The uncoordinated sites of the [Th_6_(*µ*_3_‐OH)_4_(*µ*_3_‐O)_4_(H_2_O)_6_]^12+^ SBU are occupied by six HCOO^−^ anions either in *µ*_2_‐*η* ^1^ *,η* ^1^ or *η*_1_ coordination manner. These discrete clusters are further packed into a crystalline lattice via *π–π* stackings between tpc^−^ and pba^−^ ligands (Figure 1b). In marked contrast to **Th‐103**, **Th‐104** features a 1D chain network with the *C*2/*m* symmetry, despite incorporating tpc^−^ and pba^−^ ligands as well. This observation suggests that the subtle variations in the stoichiometry of ligands can engender significant differences in metal‐ligand assembly and structural dimension. One of the most striking differences in structure between **Th‐104** and **Th‐103** is the coordination manner of pba^−^ ligand. Instead of functioning as a monotopic group in **Th‐103**, the pba^−^ in **Th‐104** interconnects two adjacent inorganic SBUs via its carboxylate and pyridine moieties, rendering it as a *µ*_2_‐*η* ^2^ *,η* ^1^ bridging ditopic linker that creates an extended 1D network extending along the *a* axis (Figure 1c). The π‐conjugated tpc^−^ ligands, however, decorate the [Th_6_(*µ*_3_‐OH)_4_(*µ*_3_‐O)_4_(H_2_O)_4_]^12+^ SBU along the *bc* plane and are efficiently separated from each other due to the spatial anchoring of pba^−^ linkers.

It is noteworthy that the coordination environments of thorium cations are surprisingly rich in these complexes, giving rise to three unprecedented hexanuclear Th_6_ SBUs (Figure 1; Figure S1, Supporting Information). Explicitly, we identified six different Th^4+^ coordination environments, among which five Th^4+^ cations (Th1, Th2, Th3, Th4, and Th6) adopt nine‐coordinated capped square antiprism geometry and one Th^4+^ (Th5) can be best described as an eight‐coordinated square antiprism (Figure S1 and Table S2, Supporting Information). Th1 is nine‐coordinated by four *µ*_3_‐O/OH groups, four carboxylate groups, and a capping H_2_O molecule, which is the prevalent coordination environment of Th^4+^ in Th‐based MOFs and clusters.^[^ ^10^ ^]^ However, zero and two water molecules can be identified in the coordination spheres of Th2 and Th3, respectively. Although Th4 is coordinated with one H_2_O molecule as well, the coordinating H_2_O is located on the vertex of the square antiprism rather than at the capping site. Moreover, Th5 is eight‐coordinated with four *µ*_3_‐O/OH groups, three carboxylate groups, and one H_2_O. Such a square antiprism geometry is common for Zr^4+^ and Hf^4+^ but rare for Th^4+^ in MOFs and clusters.^[^ ^10a^ ^]^ In addition, while Th6 exhibits a similar capped square antiprism geometry, its capping site is a N atom, donated from the pyridine moiety of the pba^−^ ligand. Such Th─N bonding is extremely rare among numerous thorium MOFs and clusters.^[^ ^18^ ^]^ This coordination diversity of Th^4+^ cation contributes to the structural complexity of the Th_6_ SBUs, engendering novel thorium clusters or coordination polymers that are unprecedented in other tetravalent cation systems.

### 2.2 Structure‐Dependent Photochromism

**Figure 2:**
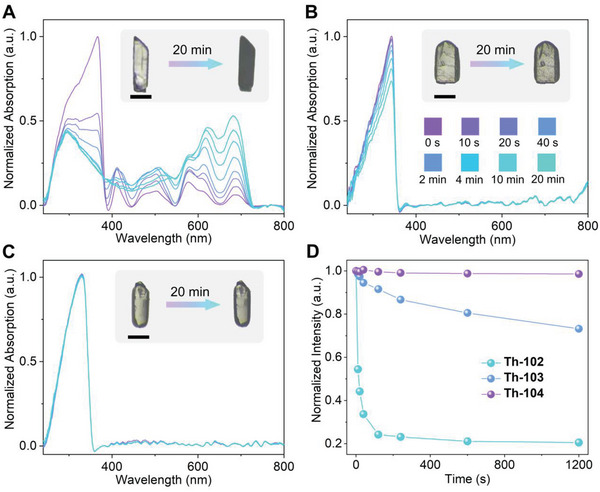
a) Time‐dependent UV–vis absorption spectra of Th‐102 in response to UV irradiation (*λ*_irr_ = 365 nm, 82.25 mJ cm^−2^). Inset: photographs showing the photochromism of Th‐102. The black scale bars in photographs represent 200 µm in length. b) Time‐dependent UV–vis absorption spectra of Th‐103 in response to UV irradiation. Inset: photographs showing the nonphotochromic property of Th‐103. c) Time‐dependent UV–vis absorption spectra of Th‐104 in response to UV irradiation. Inset: photographs showing the nonphotochromic property of Th‐104. d) Normalized UV absorption intensities of Th‐102, Th‐103, and Th‐104 as a function of radiation time.

Notably, **Th‐102** is photochromic, and the color of its crystal changes from light pink to dark green upon continuous UV irradiation (*λ*_irr_ = 365 nm, 82.25 mJ cm^−2^), which contrasts sharply with the nonphotochromic responses of **Th‐103** and **Th‐104** under the identical irradiation condition (**Figure** **2**). The time‐dependent UV–vis absorption spectra of were collected on single crystals of **Th‐102**, **Th‐103**, and **Th‐104**. As shown in Figure 2a, the nonirradiated **Th‐102** shows five sets of absorption bands with their intensities maximizing at 366, 411, 503, 578, and 687 nm. The UV absorption peak (*λ*_max_ = 366 nm) diminishes sharply in response to continuous irradiation and its absorption maximum shifts from 366 to 295 nm after approximately 4 min UV exposure. Furthermore, the intensities of the bands at 411 and 503 nm increase and reach saturation after approximately 40 s UV irradiation, while further UV irradiation results in a slight reduction in the intensities of both peaks. In contrast, the bands above 545 nm exhibit increasingly intense absorption, endowing the coloration of **Th‐102** upon continuous UV irradiation. Moreover, the photochromism of **Th‐102** is reversible and its color can be bleached by storing the crystal of **Th‐102** in the dark for 2 days (Figure S2, Supporting Information). In contrast, the spectra of **Th‐103** only exhibit one adsorption maximum at 343 nm, whose intensities decrease gradually under the identical irradiation condition. However, its absorption features in the visible region remain unchanged, in accordance with its nonphotochromic response (Figure 2b). In addition, although **Th‐104** shows a similar UV absorption band as **Th‐103**, its intensity remains approximately invariable across the dose range, making it as the most coloration‐resistant material among the three thorium complexes (Figure 2c). To quantify the response rate of coloration, the normalized intensities of the UV absorption bands as a function of radiation time were plotted as shown in Figure 2d. Obviously, **Th‐102** exhibits the most striking coloration response among the three thorium materials, as confirmed by the fastest reduction rate of absorption intensity (*λ*_max_ = 366 nm). Specifically, 120 s UV irradiation results in 75.8%, 8.4%, and 0.4% absorption reduction for **Th‐102**, **Th‐103**, and **Th‐104**, respectively, making **Th‐102** as the most sensitive UV chemosensor among three materials.

### 2.3 Structure‐Dependent Fluorochromism

**Figure 3:**
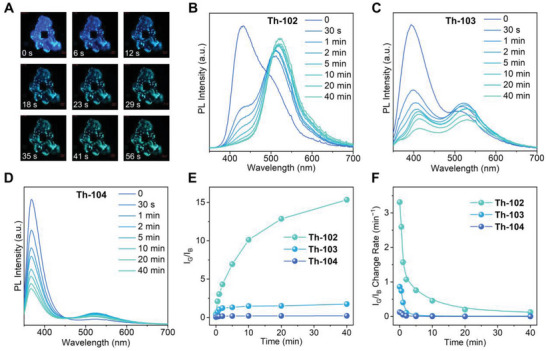
a) Photographs of Th‐102 crystals upon continuous UV irradiation. b) The time‐dependent luminescence spectra of a Th‐102 upon UV irradiation. c) The time‐dependent luminescence spectra of a Th‐103 upon UV irradiation. d) The time‐dependent luminescence spectra of a Th‐104 upon UV irradiation. e) The ratio between the excimer and monomer emission (*I*_G_/*I*_B_) as a function of radiation time for Th‐102, Th‐103, and Th‐104. f) The first‐order derivative of *I*_G_/*I*_B_ versus radiation time for Th‐102, Th‐103, and Th‐104.

More intriguingly, **Th‐102**, **Th‐103**, and **Th‐104** exhibit rather unique fluorochromism in response to UV radiation. Similar phenomena have only been observed in a limited number of materials including [(PyrO)_3_tacn]Gd(THF), Pb(_2_‐MTA)DMF, and an alkynylpyrene excimer dye (SSVV), as well as our recently reported Th‐SINAP‐100 and Th‐101.^[^ ^2^, ^8^, ^11^ ^]^ In particular, the emission color of **Th‐102** gradually switches from blue, cyan, to green upon continuous UV irradiation (**Figure** **3**a). For comparison, the fluorescence colors of **Th‐103** and **Th‐104** change from blue to cyan under identical irradiation conditions. The photoluminescence spectra of three complexes were collected from tablet samples, which were fabricated from finely ground **Th‐102**, **Th‐103**, and **Th‐104**, under 325 nm UV excitation. As shown in Figure 3b, the blue emission of **Th‐102** occurs initially as a broad band at 434 nm, which can be ascribed to the π–π* transition of tpc^−^ monomer, as evidenced by the similar emission profiles between **Th‐102** and Htpc (Figure S3, Supporting Information).^[^ ^12^ ^]^ **Th‐103** and **Th‐104**, however, display increasingly sharper monomer emission with their centers blue‐shifting toward the wavelengths of 394 and 368 nm, respectively (Figure 3c,d). Exposure of **Th‐102** to UV light (*λ*_irr_ = 365 nm, 80 mW cm^−2^) for merely 30 s results in the rapid reduction of its monomer emission and emergence of a new band centering at ≈510 nm, which can be assigned to the excimer emission from the (tpc^−^–tpc^−^) dimers (Figure 3b).^[^ ^13^ ^]^ Further UV irradiation for 20 min leads to the complete quenching of the monomer emission and enhancement of the excimer emission at 520 nm. Such a ratiometric luminescence evolution results in the switch of emission colors, which can be numerically correlated with the Commission Internationale de L'Eclairage (CIE) chromaticity coordinates (Figure S4 and Table S3, Supporting Information). Although **Th‐103** shows a similar intensity reduction of the monomer emission, its quenching rate is much slower than that of **Th‐102**. Specifically, 30 s UV exposure initiates 53.1% luminescence quenching of **Th‐103**, compare with 66.3% of **Th‐102**. Moreover, **Th‐103** exhibits a lower relative intensity of excimer emission than **Th‐102** when using the maximum monomer emission as a reference. Similar to its coloration‐resistant property, **Th‐104** shows the slowest fluorochromic response to UV. Only 24.6% luminescence reduction of the monomer emission can be resolved for **Th‐104** upon 30 s UV irradiation and its relative intensity of excimer emission is the lowest among three Th complexes as well.

Taking advantage of the ratiometric luminescence evolution in response to UV irradiation, the ratio between the excimer and monomer emission (*I*_G_/*I*_B_) was plotted as a function of radiation time to compare the UV sensitivities of these materials. As shown in Figure 3e, the *I*_G_/*I*_B_ ratios follow the trend **Th‐102** > **Th‐103** > **Th‐104** across the entire time range. Intending to obtain a systematic comparison of their response rate to UV radiation, we plotted the first‐order derivatives of *I*_G_/*I*_B_ against time. The corresponding instantaneous rates increase in the order of **Th‐102** > **Th‐103** > **Th‐104** as well (Figure 3f). These results again conjointly imply the most sensitive photo‐responsive nature of **Th‐102** among the three materials.

### 2.4 X‐Ray Dosimetry

**Figure 4:**
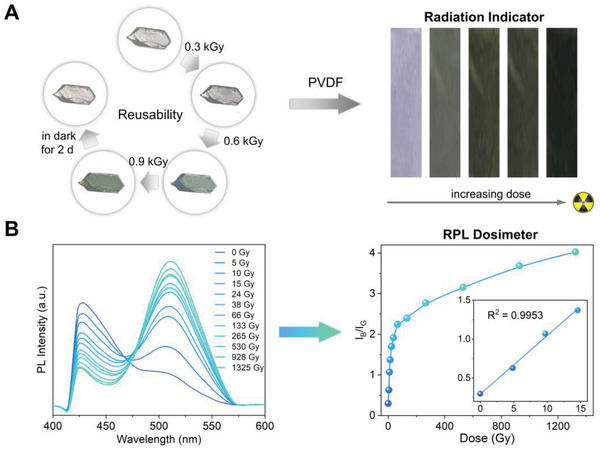
a) Micrographs of a Th‐102 sing crystal and photographs of Th‐102@PVDF showing their radiochromic responses upon X‐ray radiation. b) The dose‐dependent luminescence spectra and the ratio between the excimer and monomer emission (*I*_G_/*I*_B_) of Th‐102 as a function of X‐ray dose.

Encouraged by the sensitive colorimetric response of **Th‐102** to UV radiation, we proceeded to investigate the effects of ionizing radiation on the optical properties of **Th‐102**. Under X‐ray irradiation (W source, 60 kV, 200 µA), **Th‐102** undergoes a radiochromic transition with its crystal color gradually switching from light pink to dark green (**Figure** **4**a), which is consistent with its photochromic response to UV. In sharp contrast, both **Th‐103** and **Th‐104** are not radiochromic (Figure S5, Supporting Information). Moreover, the radiochromic transition is reversible as well, implying the excellent reusability of **Th‐102**. The radiochromism can be utilized for semi‐quantitative or at least qualitative detection of X‐ray, likely the widely used radiation indicator. Furthermore, the **Th‐102** crystals were fabricated with polyvinylidene fluoride (PVDF) into a strip to facilitate the practical application.^[^ ^14^ ^]^ Gratifyingly, **Th‐102** crystals impart radiochromism to the afforded **Th‐102**@PVDF strip, which could be further used as a test kit for onsite analysis. This type of colorimetric dosimeters offers significant benefits of low cost and ease of use, which are favorable for certain scenarios where these attributes take precedence over precise quantification of radiation dose.^[^ ^15^ ^]^

Besides radiochromism, similar fluorochromic transitions from blue to cyan to green occur for **Th‐102**, **Th‐103**, and **Th‐104** upon X‐ray irradiation, rendering them as another rare examples of radio‐photoluminescence (RPL) materials based on organic–inorganic hybrids (Figure S6, Supporting Information).^[^ ^2^, ^8^ ^]^ The X‐ray dose‐dependent luminescence spectra were collected on the tablet samples of **Th‐102**, **Th‐103**, and **Th‐104** under 365 nm UV excitation. As depicted in Figure S6a–c (Supporting Information), the monomer emissions decrease while the excimer emissions increase upon X‐ray irradiation with increasing dose for **Th‐102**, **Th‐103**, and **Th‐104**. However, the rates of change in both emissions vary among the three complexes. To conduct an assessment of their sensitivities to X‐ray radiation, the ratios between the excimer and monomer emission were plot as a function of X‐ray dose (Figure S6d, Supporting Information). The *I*_G_/*I*_B_ ratios follow the trend **Th‐102** > **Th‐103** > **Th‐104** across the entire dose range, whose tendency agrees well with that of UV‐induced fluorochromic responses. These observations, in conjunction with the different radiochromic behaviors, suggest that **Th‐102** features the highest degree of X‐ray sensitivity among the three thorium complexes.

To more accurately establish a quantitative correlation between *I*_G_/*I*_B_ and X‐ray dose for **Th‐102**, its real‐time luminescence spectra in response to increasing X‐ray dose were collected on a single crystal of **Th‐102** using a solid‐state microspectrophotometer under 365 nm UV excitation, which generates luminescence in a more efficient manner (Figure S7, Supporting Information). As shown in Figure 4b. *I*_G_/*I*_B_ increases sharply at the low dose region and gradually approaches the saturation point upon further irradiation. Notably, excellent linear relationship (*R* ^2^ = 0.9953) of *I*_G_/*I*_B_ versus X‐ray dose can be obtained from 0 to 15 Gy, making accurate X‐ray dosimetry at the therapeutic level (0.5–10 Gy) feasible.^[^ ^2e^ ^]^ It is worth noting that the linear range of **Th‐102** is broader than those of a plasmonic nanosensor gel (0–3 Gy) and a gel nanosensor (0–4 Gy).^[^ ^2a,e^ ^]^ Moreover, linear regression analysis of the monomer emission was performed to calculate the limit of detection (LOD). The LOD of X‐ray was derived from 3*σ*/slope and determined to be 1.5 mGy, representing a significant improvement in terms of sensitivity compared to Th‐SINAP‐100 and Th‐101 (Figure S8, Supporting Information).^[^ ^8b,c^ ^]^ Moreover, **Th‐102** features a lower LOD than Htpbz@Th‐SINAP‐2, which is one of the most sensitive radiochromic coordination polymers.^[^ ^16^ ^]^

### 2.5 Radiolytic Stability

**Figure 5:**
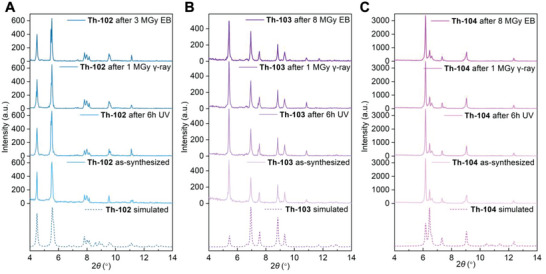
Powder X‐ray diffraction patterns of a) Th‐102, b) Th‐103, and c) Th‐104 before and after UV, γ‐ray, or electron beam (EB) irradiation.

As one of the most critical properties of dosimeters, the radiolytic stabilities of **Th‐102**, **Th‐103**, and **Th‐104** were comparatively evaluated via powder X‐ray diffraction (PXRD) and Fourier transform infrared spectroscopy (FTIR). As shown in **Figure** **5** and Figures S9–S11 (Supporting Information), no crystallographic or spectroscopic evidence of decomposition can be observed in all three materials after being exposed to UV radiation (80 mW cm^−2^) for 6 h. Furthermore, when irradiated with 1 MGy γ‐ray dose (^60^Co source, 2.22 × 10^15^ Bq), all complexes exhibited no occurrence of breakdown, suggesting their excellent radiolytic stabilities. Moreover, **Th‐104** can withstand an accumulated dose of 8 MGy electron beam (EB) provided by an electron cyclotron (1.2 MeV), rendering it as one of the most radiation‐resistant organic–inorganic hybrids reported to date (Table S4, Supporting Information).^[^ ^17^ ^]^ For comparison, **Th‐102** exhibited diminished crystallinity under the comparable irradiation condition (Figure S12, Supporting Information). However, its structure remained intact when the dose decreased to 3 MGy (Figure 5a), suggesting that **Th‐102** can undoubtedly withstand the radiation at the therapeutic level. The high radiolytic stabilities of these thorium complexes can be attributed to the robust coordination bonds between Th_6_ SBUs and the carboxylate groups, as well as the ligand aromaticity that stabilizes the organic moieties via *π–π* interactions.^[^ ^17^, ^18^ ^]^ These inherent structural characteristics result in excellent moisture and thermal stabilities. The structures of **Th‐102**, **Th‐103**, and **Th‐104** remain intact when subjected to ambient relative humidity (RH) conditions (55% and 95%) for 24 h (Figures S13–S15, Supporting Information). In addition, thermogravimetric analysis (TGA) indicates that **Th‐102**, **Th‐103**, and **Th‐104** are stable up to approximately 140, 150, 260 °C, respectively (Figure S16, Supporting Information).

### 2.6 Mechanism

**Figure 6:**
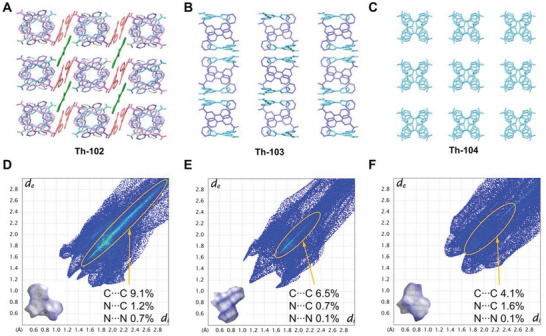
Representations showing the intermolecular packing of tpc^−^ ligands in a) Th‐102, b) Th‐103, and c) Th‐104. Hirshfeld surfaces and the 2D‐fingerprint plots of the selected tpc^−^ ligands in d) Th‐102, e) Th‐103, and f) Th‐104.

To probe mechanistic features of the different photochromic and fluorochromic responses, we first ruled out the possible changes of structures and compositions of **Th‐102**, **Th‐103**, and **Th‐104** upon irradiation by PXRD (Figure 5) and FTIR (Figures S9–S11, Supporting Information), respectively. There is precedence in literatures that the modulation of intermolecular interactions of stimuli‐responsive ligands can lead to difference in electron transfer (ET) pathways and consequently modify their optical properties, as illustrated in a varieties systems including viologen, spiropyran, flavylium, etc.^[^ ^19^ ^]^ Therefore, we speculated that the difference photochromic and fluorochromic responses can be pertinent to the diverse degrees of intermolecular *π–π* interactions of the tpc^−^ ligands. To verify this assumption, a more detailed comparison of the molecular packings in **Th‐102**, **Th‐103**, and **Th‐104** was conducted. As shown in **Figure** **6**a, **Th‐102** features the most densely packed arrangement of ligands that are exclusively composed of tpc^−^ moieties. Specifically, six crystallographically independent tpc^−^ ligands can be identified, which offer a variety of ET pathways between the neighboring tpc^−^ moieties. For **Th‐103**, two crystallographically unique tpc^−^ can be resolved, giving rise to two different ET manners between the adjacent tpc^−^ groups (Figure 6b). In contrast, only one crystallographically independent tpc^−^ ligand can be found in **Th‐104**, enabling the ET least feasible (Figure 6c). To delve deeper into the different degrees of intermolecular interactions, Hirshfeld surface analysis on tpc^−^ ligands in three complexes was performed using *CrystalExplorer 17.5*.^[^ ^20^ ^]^ Figure 6d–f represents the 2D‐fingerprint plots and the Hirshfeld surfaces of selected tpc^−^ ligands, which have the closest tpc^−^···tpc^−^ distances in their respective structures. The 2D‐fingerprint plots indicate that the C C, C N, and N N contacts of tpc^−^, which mainly arise from the intermolecular *π–π* interactions, account for 11%, 7.3%, and 5.8% to the total Hirshfeld surfaces for **Th‐102**, **Th‐103** and **Th‐104**, respectively (Figures S17–S19, Supporting Information). Therefore, the intermolecular *π–π* interactions of the tpc^−^ ligands follow the tendency of **Th‐102** > **Th‐103** > **Th‐104**, making the intermolecular ET and correspondingly photochromic response most accessible for **Th‐102**. Indeed, paramagnetic resonance (EPR) spectrum study revealed the presence of radical species in the spectrum of irradiated **Th‐102**, while both **Th‐103** and **Th‐104** exhibited neglectable resonance signals under comparable irradiation conditions (Figure S20, Supporting Information). The generation of radical species in **Th‐102** is anticipated due to the close tpc^−^···tpc^−^ intermolecular distance and correspondingly an accessible ET pathway, which has been proved to account for the photochromism or radiochromism of pyridine‐based of materials.^[^ ^8^, ^21^ ^]^ In a similar vein, promoting the tpc^−^ moieties to the excited state and affording (tpc^−^···tpc^−^)* excimers are more accessible in **Th‐102** due to the most facile intermolecular ET pathway of tpc^−^ ligands.^[^ ^13^, ^22^ ^]^ Consequently, **Th‐102** features the most sensitive photo‐ or radio‐responsive properties, making it as the most sensitive UV and X‐ray sensor among the three materials.

## 3 Conclusion

In summary, we have for the first time introduced control over the ligand ratios between a stimuli‐responsive ligand and an auxiliary unit in organic–inorganic hybrids, enabling the regulation of their sensitivities to UV and X‐ray irradiation. By preparing **Th‐102** as a mono‐ligand benchmark material, we have demonstrated that introducing an ancillary ligand produces two mixed‐ligand organic–inorganic hybrids (**Th‐103** and **Th‐104**) instead of forming separate domains. We assessed their photochromic and fluorochromic responses to UV irradiation, revealing a positive correlation between UV sensitivity and the molar ratio of the stimuli‐responsive tpc^−^ ligand versus pba^−^ ligand, following the trend of **Th‐102** > **Th‐103** > **Th‐104**. Moreover, SCXRD and EPR studies have shown that the intermolecular interactions of stimuli‐responsive ligands can be modulated by the mixed‐ligand strategy, leading to the manipulation of electron transfer (ET) pathways and modification of their sensitivities. **Th‐102**, which features the most densely packed tpc^−^ moieties and the strongest tpc^−^···tpc^−^ intermolecular interaction, exhibits an extremely low detection limit (1.5 mGy) toward X‐ray. These results should aid in understanding the structure‐property correlation of stimuli‐responsive materials and also give a new direction to fine‐tune the sensitivity of radiation detection materials.

[CCDC 2283872–2283874 contain the supplementary crystallographic data for this paper. These data can be obtained free of charge from The Cambridge Crystallographic Data Centre via https://www.ccdc.cam.ac.uk/data_request/cif.]

## Conflict of Interest

The authors declare no conflict of interest.

## Supporting information

Supporting Information

Supporting Information

## Data Availability

The data that support the findings of this study are available from the corresponding author upon reasonable request.
