# Supplementary material for: Fine‐Tuning X‐Ray Sensitivity in Organic–Inorganic Hybrids via an Unprecedented Mixed‐Ligand Strategy
Source: Adv Sci (Weinh). 2023 Nov 8;11(1):2305378. doi: 10.1002/advs.202305378 (PMC10767407; doi:10.1002/advs.202305378)
Supplement: Supplementary file 1 — Supporting Information [file ADVS-11-2305378-s002.pdf]

ADVANCED SCIENCE
Open Access

Supporting Information

for *Adv. Sci.*, DOI 10.1002/advs.202305378

Fine-Tuning X-Ray Sensitivity in Organic–Inorganic Hybrids via an Unprecedented Mixed-Ligand Strategy

*Huangjie Lu, Zhaofa Zheng, Huiliang Hou, Yaoyao Bai, Jie Qiu, Jian-Qiang Wang and Jian Lin**

Supporting Information

Fine-Tuning X-ray Sensitivity in Organic-Inorganic hybrids via an Unprecedented Mixed-Ligand Strategy

*Huangjie Lu, Zhaofa Zheng, Huiliang Hou, Yaoyao Bai, Qiu Jie, Jian-Qiang Wang, and Jian Lin**

S1. EXPERIMENTAL SECTION

Materials and Synthesis

Caution! *Caution! Th-232 used in this study is an emitter with the daughter of radioactive Ra-228. All of the thorium compounds used and investigated were operated in an authorized laboratory designed for actinide element studies. Standard protections for radioactive materials should be followed.*

Materials. $Th(NO_3)_4·6H_2O$ (99.9%, Changchun Institute of Applied Chemistry, Chinese Academy of Sciences), 2,2':6',2"-terpyridine-4'-carboxylic acid (HTPC) (99%, Jilin Chinese Academy of Sciences - Yanshen Technology Co., Ltd), 3-(pyridin-4-yl)benzoic acid (Hbpa) (98%, Jilin Chinese Academy of Sciences - Yanshen Technology Co., Ltd), DMF (99.5%, Aladdin), $HClO_4$ (AR, ≥99.5%, Sinopharm Chemistry Reagent Co., Ltd), and CH_3CH_2COOH (AR, ≥99.5%, Sinopharm Chemistry Reagent Co., Ltd) were used as received from commercial suppliers without further purification.

Synthesis. A mixture of $Th(NO_3)_4·6H_2O$ (11.6 mg, 0.02 mmol), $HClO_4$ (40 μL), HTPC (5.5 mg, 0.02 mmol), DMF (0.125 mL), and deionized water (0.875 mL) were loaded into a 5 mL vials. The vial was sealed and heated to 100 °C for 48 h and then cooled to room temperature under ambient condition (**Th-102**). A mixture of $Th(NO_3)_4·6H_2O$ (5.9 mg, 0.01 mmol), $HClO_4$ (30 μL), HTPC (2.8 mg, 0.01 mmol), Hbpa (2 mg, 0.01 mmol), DMF (0.2 mL), and deionized water (0.8 mL) were loaded into a 5 mL vials (**Th-103**). A mixture of $Th(NO_3)_4·6H_2O$ (5.9 mg, 0.01 mmol), CH_3COOH (5 μL), CH_3CH_2COOH (50 μL), HTPC (2.8 mg, 0.01 mmol), Hbpa (2 mg, 0.01 mmol), DMF (0.2 mL), and deionized water (0.8 mL) were loaded into a 5 mL vials (**Th-104**). The vial was sealed and heated to 100 °C for 48 h (**Th-102** and **Th-104**) or 120 h (**Th-103**) and then cooled to room temperature under ambient

condition. Colorless block crystals of **Th-102**, and **Th-104** ,and pink block crystals of **Th-103** were isolated after being washed with ethanol and allowed to air-dry at room temperature. The yield of **Th-102** was calculated to be 52.6(5)% based on HTPC, **Th-103** and **Th-104** was calculated to be 22.4(3)% and 72.8(5)% based on $Th(NO_3)_4 \cdot 6H_2O$, respectively.

Characterizations

Crystallographic Analysis. Single crystal X-ray diffraction measurements were performed using a Bruker D8-Venture single crystal X-ray diffractometer equipped with an IμS 3.0 microfocus X-ray source (Mo–Kα radiation, λ = 0.71073Å) and a CMOS detector at 298 K. The data frames were collected using the program APEX3 and processed using the program SAINT routine in APEX3. The structures were solved by the direct method and refined on F^2 by full-matrix least-squares methods using SHELXTL-2014 program.[1] Powder X-ray diffraction (PXRD) data were collected from 5 to 50° with a step of 0.02° and the time for data collection was 0.5 s on a Bruker D8 Advance diffractometer with Cu Kα radiation (λ=1.54056 Å) and a Lynxeye one-dimensional detector.

UV-Vis and Photoluminescence Spectroscopy. The solid-state UV-Vis absorption of single crystals of **Th-102**, **Th-103**, and **Th-104** were recorded on a Craic Technologies microspectrophotometer. Crystals were placed on quartz slide, and data was collected after auto-set optimization. The photoluminescence spectra of bulk samples were collected on an Edinburgh Instruments FS5 steady state spectrofluorometer with 325 nm UV excitation. The real-time photoluminescence spectra of a single crystal of **Th-102** in response to X-ray were recorded on a Craic Technologies microspectrophotometer. When the 365 nm excitation light was selected, an optical filter masking signal below 420 nm was applied in order to mask the interference of excitation light. The sources of X-ray were provided by a W Kα radiation source (60 kV, 12W). The decay curves of bulk samples were collected on an Edinburgh Instruments FLS 980 spectrometer. PL quantum-yield (PLQY) was recorded using a HORIBA scientific Fluorolog-3 spectrophotometer with a quantum-yield accessory.

Fourier Transform Infrared Spectroscopy. The IR spectra were recorded using a FTIR spectrometer (Thermo Nicolet 6700 spectrometer) equipped with a diamond attenuated total reflectance (ATR) accessory in the range of 400–4000 cm^{-1}.

Electron Paramagnetic Resonance (EPR) Study. The EPR measurements were performed on a JEOL-FA200 spectrometer at X-band with 100-kHz field modulation. The EPR spectra of nonirradiated and irradiated samples were recorded at room temperature and the microwave power used was 1.0 mW.

Radiolytic Stability. The radiation resistance of **Th-102**, **Th-103**, and **Th-104** were examined by irradiating the powdery sample with UV, EB, or γ-ray irradiation. The sources of UV, EB, and γ-ray were provided by a LED light (365 nm, 25 W), an electron accelerator (1.2 MeV), and a ^{60}Co irradiation source (2.22×10^{15} Bq), respectively. **Th-102**, **Th-103**, and **Th-104** were irradiated with accumulated doses with dose rates of 80 mW cm^{-2}, 150 kGy h^{-1}, and 11.8 kGy h^{-1} for UV, EB, γ-ray, respectively. PXRD and FTIR analyses on the irradiated samples were performed to confirm the radiation resistance of **Th-102**, **Th-103**, and **Th-104.**

Hirshfeld Surface Analysis

The SC-XRD structures of **Th-102**, **Th-103**, and **Th-104** was directly used in the Hirshfeld surface analysis. Hirshfeld surfaces of the selected tpc^{-} ligands were calculated in CrystalExplorer17.5. The two-dimensional fingerprint plots were generated as shown in Figure S10 to S12.

S2. FIGURES AND TABLES

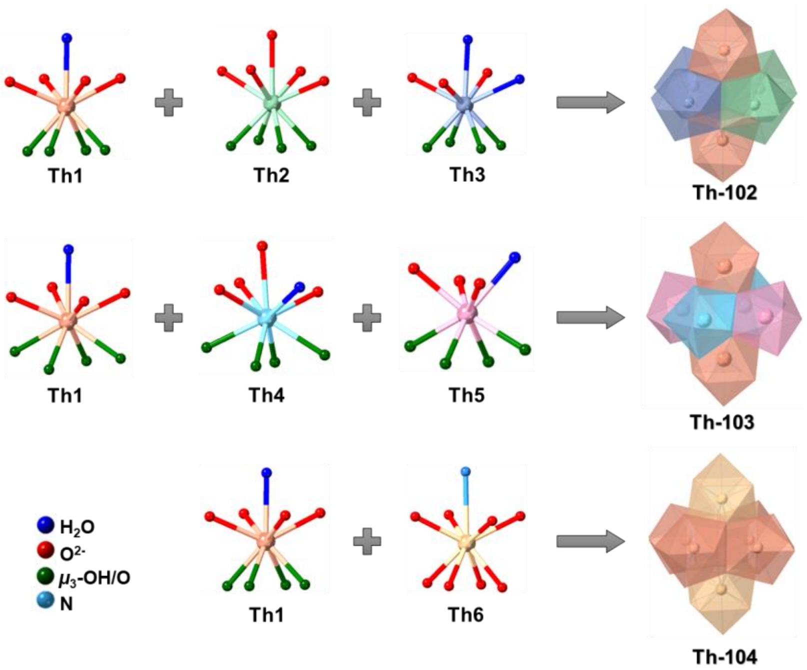

Figure S1. Coordination environment of all the Th cations and hexameric $[Th_6(OH)_4O_4(H_2O)_6]^{12+}$/$[Th_6(OH)_4O_4(H_2O)_4]^{12+}$ cores.

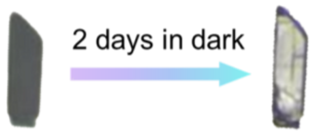

Figure S2. The bleaching process by storing the crystal of **Th-102** in dark for 2 days.

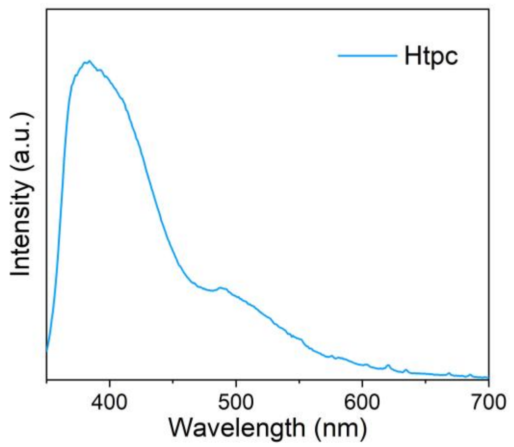

Figure S3. The photoluminescence spectrum of Htpc under 325 nm UV excitation.

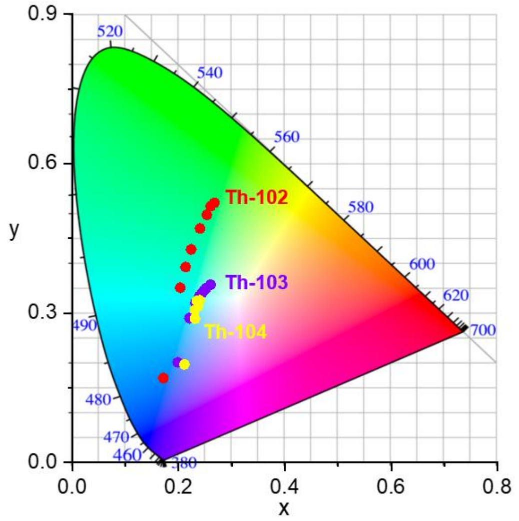

Figure S4. Evolution of the CIE chromaticity coordinates of **Th-102**, **Th-103**, and **Th-104** with increasing UV irradiation doses (0 – 192 J cm^{-2}).

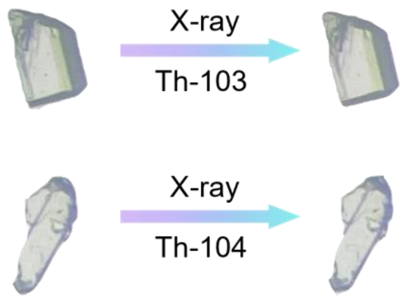

Figure S5. The micrographs of **Th-103** and **Th-104** before and after X-ray irradiation.

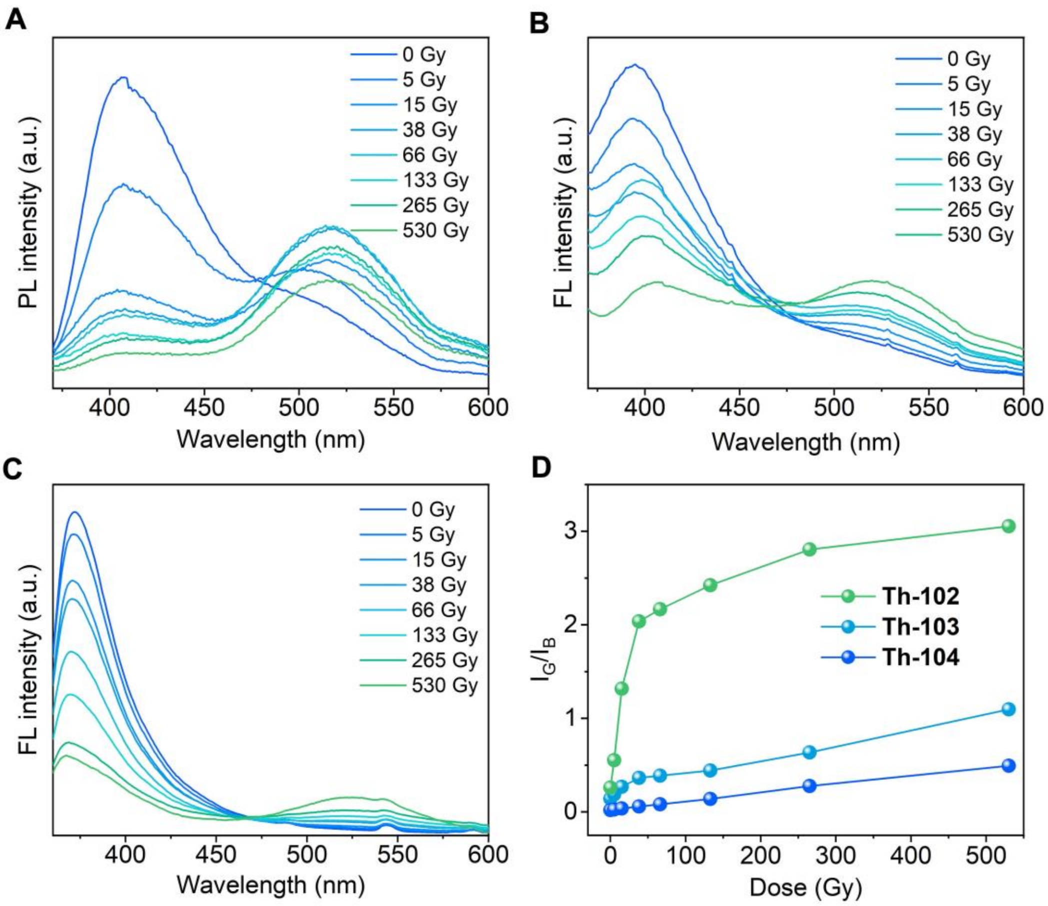

Figure S6. (a) The time-dependent luminescence spectra of a **Th-102** upon X-ray irradiation. (b) The time-dependent luminescence spectra of a **Th-103** upon X-ray irradiation. (c) The time-dependent luminescence spectra of a **Th-104** upon X-ray irradiation. (d) The ratio between the excimer and monomer emission (I_G/I_B) as a function of X-ray dose for **Th-102**, **Th-103**, and **Th-104**.

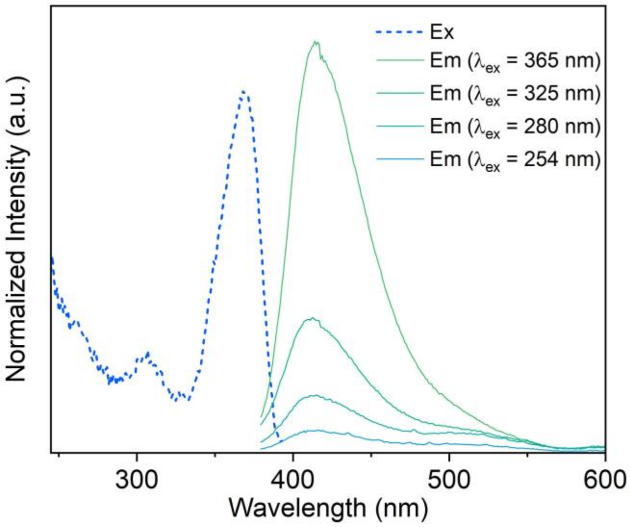

Figure S7. The excitation and wavelength-dependent luminescence spectra of a **Th-102**.

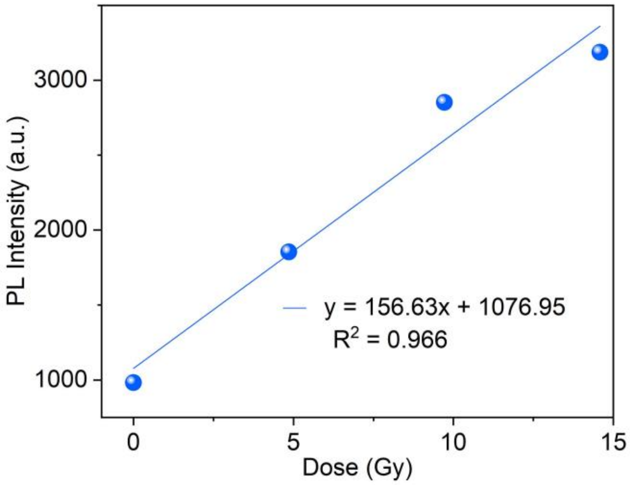

Figure S8. The linear correlation between the luminescence intensity at 510 nm and the radiation dose in low dose range. The linear domain in low dose range can be fitted as y = 156.63 x + 1076.95 where y is the luminescence intensity at 510 nm and x is the radiation dose. The standard deviation (σ) is the standard error of the luminescence measurement, as determined by the baseline measurement of blank samples at 510 nm. If defining three times of the standard deviation as the detectable signal, the detection limit can be projected as 3σ/slope = 3 × 0.07931 /156.63 = 1.5 mGy.

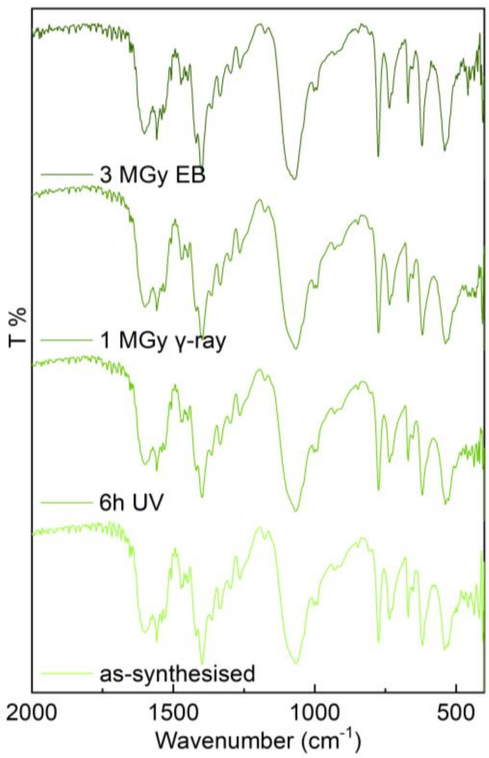

Figure S9. The FTIR spectra of **Th-102** before and after irradiation with UV, γ-ray, and EB radiation.

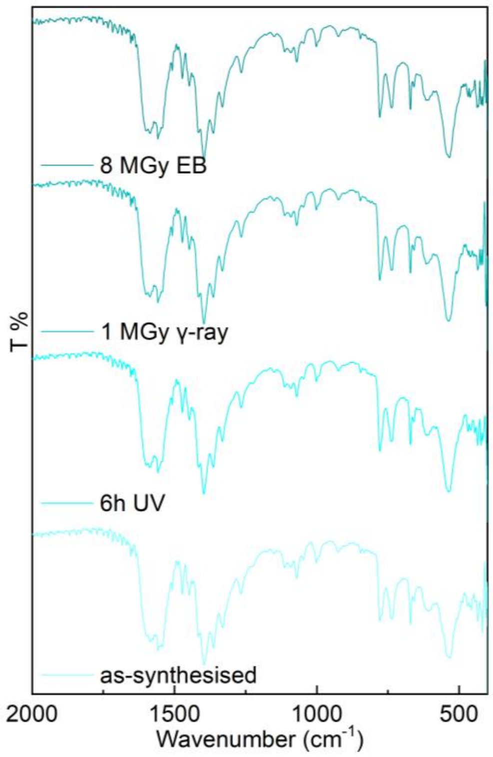

Figure S10. The FTIR spectra of **Th-103** before and after irradiation with UV, γ-ray, and EB radiation.

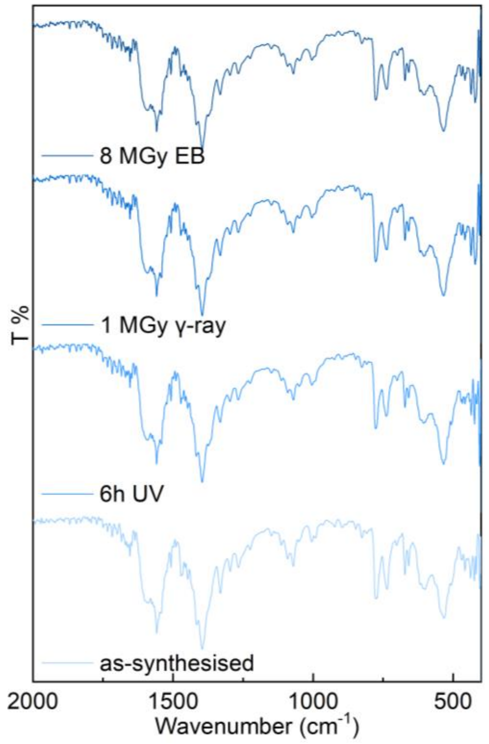

Figure S11. The FTIR spectra of **Th-104** before and after irradiation with UV, γ-ray, and EB radiation.

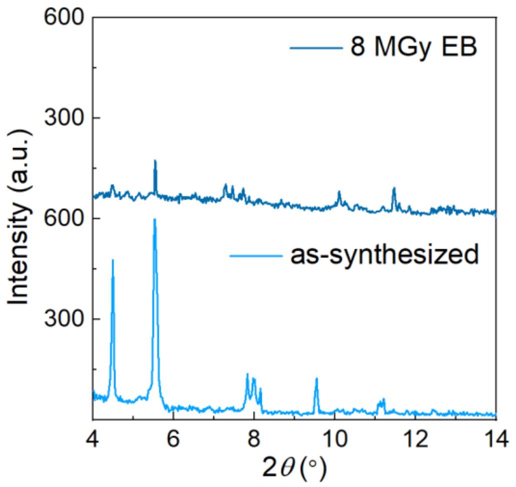

Figure S12. The PXRD patterns of **Th-102** before and after 8 MGy electron beam (EB) irradiation.

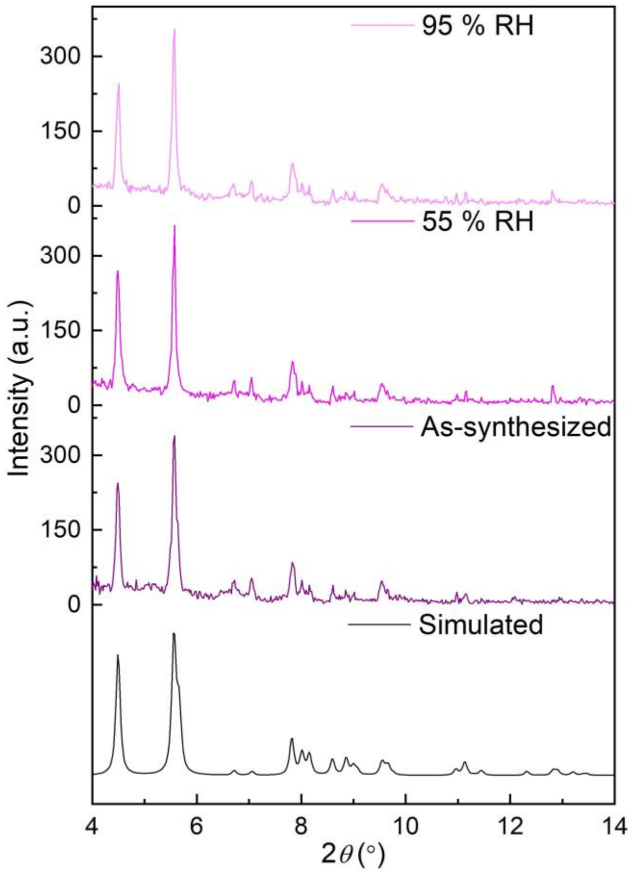

Figure S13. Powder X-ray diffraction patterns of **Th-102** treated with different RH conditions.

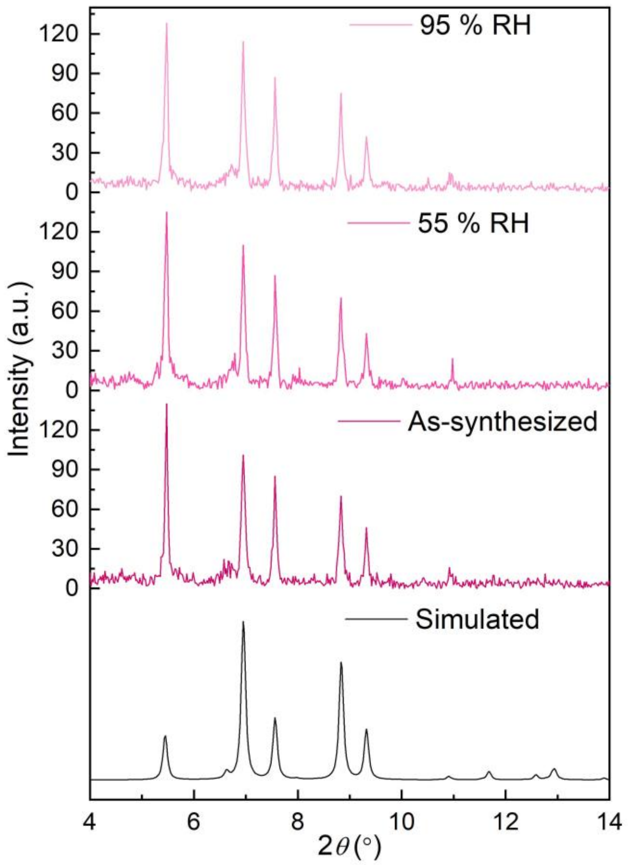

Figure S14. Powder X-ray diffraction patterns of **Th-103** treated with different RH conditions.

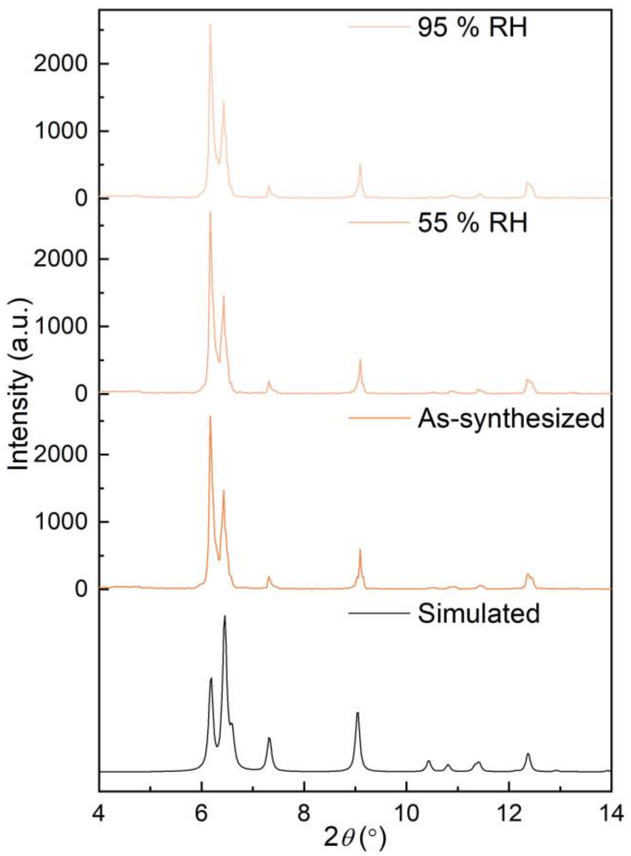

Figure S15. Powder X-ray diffraction patterns of **Th-104** treated with different RH conditions.

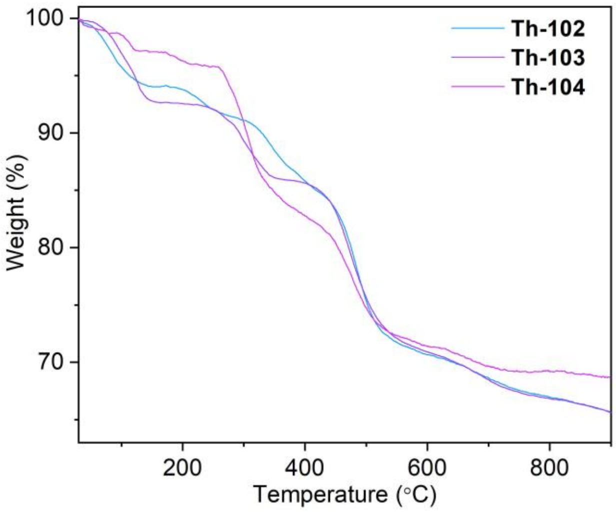

Figure S16. Thermogravimetric analysis curves of **Th-102**, **Th-103**, and **Th-104**.

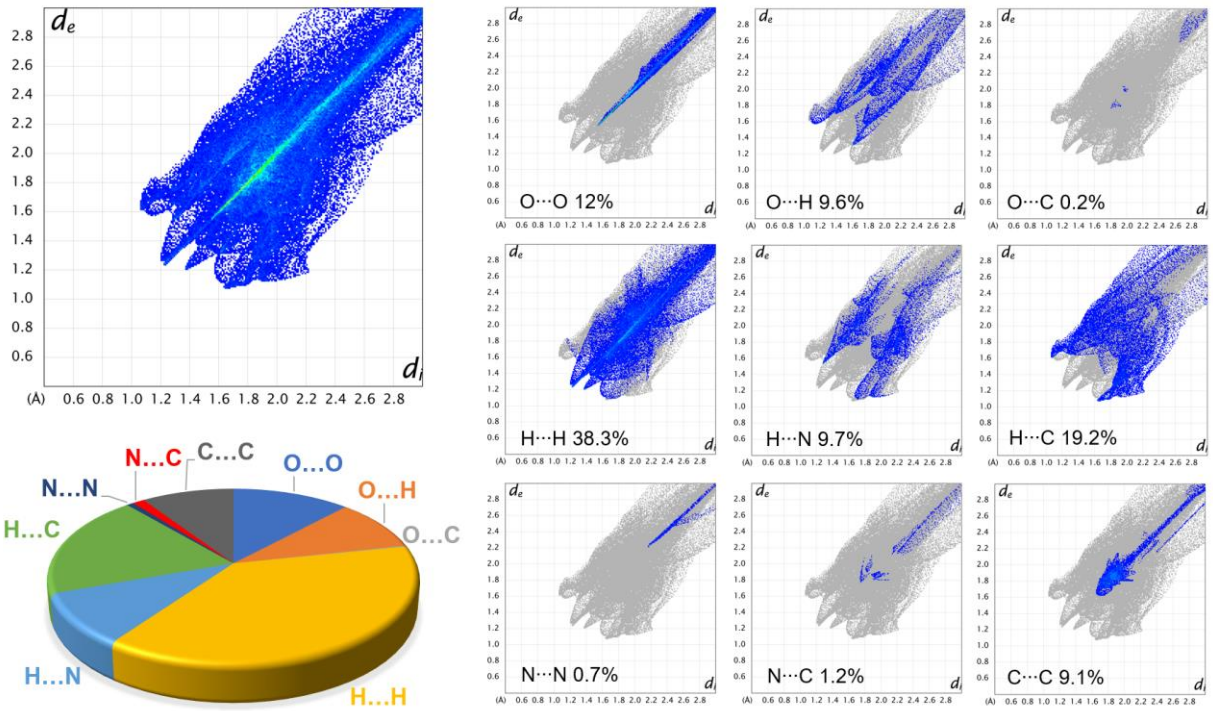

Figure S17. The fingerprint plots and relative contributions of different intermolecular contacts to the Hirshfeld surface areas for **Th-102**.

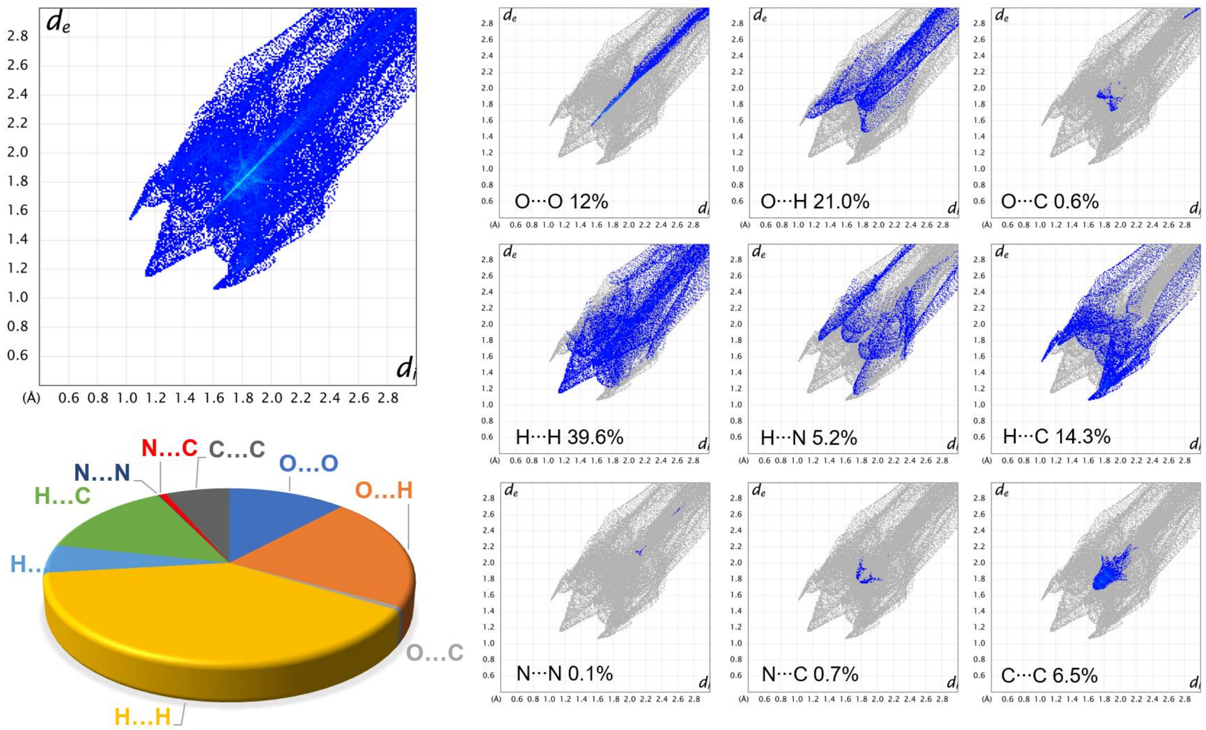

Figure S18. The fingerprint plots and relative contributions of different intermolecular contacts to the Hirshfeld surface areas for **Th-103**.

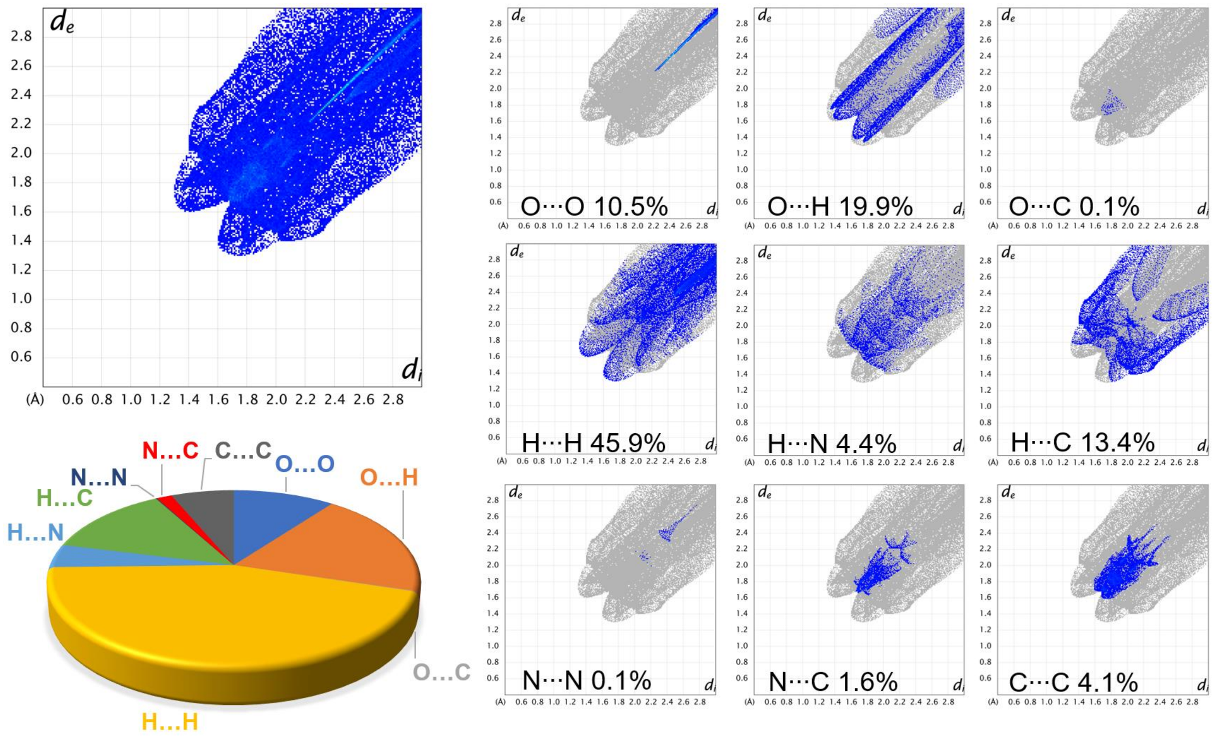

Figure S19. The fingerprint plots and relative contributions of different intermolecular contacts to the Hirshfeld surface areas for **Th-104**.

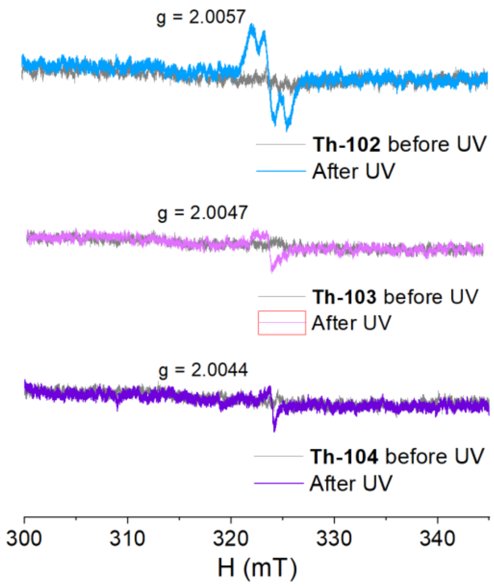

Figure S20. The EPR spectra of nonirradiated, UV and irradiated of **Th-102**, **Th-103**, and **Th-104**.

Table S1. Crystallographic data of **Th-102**, **Th-103**, and **Th-104**.

Sample	**Th-102**	**Th-103**	**Th-104**
Mass	4856.44	3600.05	3745.61
Color	Pink	Colorless	Colorless
Habit	Block	Block	Block
Space group	*C*2/*c*	*Pccn*	*C*2/*m*
a (Å)	31.7400(14)	15.1500(5)	17.191(1)
b (Å)	25.0234(10)	23.3651(8)	28.528(1)
c (Å)	26.3104(11)	32.4213(8)	14.7593(8)
α (°)	90	90	90
β (°)	91.752(2)	90	114.984(7)
γ (°)	90	90	90
V (Å^3)	20887.1(15)	11476.5(6)	6561.0(7)
Z	4	4	2
T (K)	273(2)	297(2)	297(2)
λ (Å)	0.71073	0.71073	0.71073
Max 2θ (°)	50.000	49.996	50.000
ρ_{calcd} (g cm^{-3})	1.544	2.084	1.896
μ (Mo Ka)	0.71073	0.71073	0.71073
R_1	0.0440	0.0556	0.0372
wR_2	0.1220	0.1596	0.1007
R_{int}	0.1202	0.0736	0.0653
GOF	1.004	1.127	1.032

Table S2. The coordination number and bond length of the Th^{4+} cations in **Th-102**, **Th-103**, and **Th-104**.

	Metal center	Coord. no.	$d_{\mu3\text{-OH/O}}$ (Å)	d_{tpc/COO^-} (Å)	d_{H2O} (Å)	d_N (Å)
**Th-102**	Th1	9	2.276~2.550	2.464~2.495	2.589	/
	Th2	9	2.254~2.530	2.470~2.711	/	/
	Th3	9	2.269~2.541	2.481~2.521	2.607~2.641	/
**Th-103**	Th1	9	2.252~2.595	2.455~2.502	2.718~2.880	/
	Th4	9	2.292~2.525	2.447~2.624	2.495	/
	Th5	8	2.283~2.491	2.378~2.508	2.491	/
**Th-104**	Th1	9	2.245~2.546	2.437~2.522	2.631~2.818	/
	Th6	9	2.311~2.513	2.445~2.511	/	2.829

Table S3. The CIE chromaticity coordinates (x, y) of photoluminescence upon irradiation with different UV dose for **Th-102**, **Th-103**, and **Th-104**.

Dose ($J\cdot cm^{-2}$)	**Th-102** (x, y)	**Th-103** (x, y)	**Th-104** (x, y)
0	(0.171,0.169)	(0.198,0.201)	(0.211,0.197)
2.4	(0.203,0.351)	(0.220,0.290)	(0.231,0.289)
4.8	(0.213,0.392)	(0.231,0.320)	(0.237,0.314)
9.6	(0.224,0.428)	(0.239,0.333)	(0.239,0.325)
24	(0.240,0.470)	(0.244,0.332)	(0.239,0.326)
48	(0.253,0.497)	(0.248,0.338)	(0.238,0.323)
96	(0.260,0.514)	(0.251,0.336)	(0.234,0.315)
192	(0.267,0.521)	(0.260,0.357)	(0.231,0.307)

Table S4. Comparison of the radiation stabilities of organic-inorganic hybrids.

Material	EB irradiation	γ irradiation	Reference
**Th-102**	**3 MGy**	**1 MGy**	**This work**
**Th-103**	**8 MGy**	**1 MGy**	**This work**
**Th-104**	**8 MGy**	**1 MGy**	**This work**
Th-101	6 MGy	3 MGy	[2]
Th-SINAP-100	1 MGy	1 MGy	[3]
M-UiO-66 (M = Zr, Ce, Hf, Th, and Pu)	N/A	3MGy	[4]
TOF-16	N/A	4 MGy	[5]
$[(CH_3)_2NH_2][UO_2(L1)]$	200 kGy	200 kGy	[6]
FJSM-InMOF	100 kGy	200 kGy	[7]
$[Ni_3Th_6(\mu_3\text{-})_4(\mu_3OH)_4(IN)_{12})(H_2O)_{12}]$ $\cdot(OH)_6\cdot5DMF\cdot2H_2O$	400 kGy	100 kGy	[8]
SCU-100	200 kGy	200 kGy	[9]
SCU-101	200 kGy	200 kGy	[10]
SCU-200	200 kGy	200 kGy	[11]
MIL-100(Al)	N/A	2 MGy	[12]

S3. SUPPLEMENTARY REFERENCES

[1] G. M. Sheldrick, *Acta Crystallogr. Sect. A* **2015**, *71*, 3.
[2] H. Lu, H. Hou, Y.-C. Hou, Z. Zheng, Y. Ma, Z. Zhou, X. Guo, Q.-J. Pan, Y. Wang, Y. Qian, J.-Q. Wang, J. Lin, *J. Am. Chem. Soc.* **2022**, *144*, 3449.
[3] H. Lu, J. Xie, X.-Y. Wang, Y. Wang, Z.-J. Li, K. Diefenbach, Q.-J. Pan, Y. Qian, J.-Q. Wang, S. Wang, J. Lin, *Nat. Commun.* **2021**, *12*, 2798.
[4] A. M. Hastings, M. Fairley, M. C. Wasson, D. Campisi, A. Sarkar, Z. C. Emory, K. Brunson, D. B. Fast, T. Islamoglu, M. Nyman, P. C. Burns, L. Gagliardi, O. K. Farha, A. E. Hixon, J. A. LaVerne, *Chem. Mater.* **2022**, *34*, 8403.
[5] S. E. Gilson, M. Fairley, P. Julien, A. G. Oliver, S. L. Hanna, G. Arntz, O. K. Farha, J. A. LaVerne, P. C. Burns, *J. Am. Chem. Soc.* **2020**, *142*, 13299.
[6] Y. Wang, Z. Liu, Y. Li, Z. Bai, W. Liu, Y. Wang, X. Xu, C. Xiao, D. Sheng, J. Diwu, J. Su, Z. Chai, T. E. Albrecht-Schmitt, S. Wang, *J. Am. Chem. Soc.* **2015**.
[7] Y.-J. Gao, M.-L. Feng, B. Zhang, Z.-F. Wu, Y. Song, X.-Y. Huang, *J. Mater. Chem. A* **2018**, *6*, 3967.
[8] H. Xu, C.-S. Cao, H.-S. Hu, S.-B. Wang, J.-C. Liu, P. Cheng, N. Kaltsoyannis, J. Li, B. Zhao, *Angew. Chem. Int. Ed.* **2019**, *58*, 6022.
[9] D. Sheng, L. Zhu, C. Xu, C. Xiao, Y. Wang, Y. Wang, L. Chen, J. Diwu, J. Chen, Z. Chai, T. E. Albrecht-Schmitt, S. Wang, *Environ. Sci. Technol.* **2017**, *51*, 3471.
[10] L. Zhu, D. Sheng, C. Xu, X. Dai, M. A. Silver, J. Li, P. Li, Y. Wang, Y. Wang, L. Chen, C. Xiao, J. Chen, R. Zhou, C. Zhang, O. K. Farha, Z. Chai, T. E. Albrecht-Schmitt, S. Wang, *J. Am. Chem. Soc.* **2017**, *139*, 14873.
[11] H. Liu, H. Qin, N. Shen, S. Yan, Y. Wang, X. Yin, X. Chen, C. Zhang, X. Dai, R. Zhou, X. Ouyang, Z. Chai, S. Wang, *Angew. Chem. Int. Ed.* **2020**, *59*, 1.
[12] C. Volkringer, C. Falaise, P. Devaux, R. Giovine, V. Stevenson, F. Pourpoint, O. Lafon, M. Osmond, C. Jeanjacques, B. Marcillaud, J. C. Sabroux, T. Loiseau, *Chem. Commun.* **2016**, *52*, 12502.
