# Supplementary material for: Fine‐Tuning X‐Ray Sensitivity in Organic–Inorganic Hybrids via an Unprecedented Mixed‐Ligand Strategy
Source: Adv Sci (Weinh). 2023 Nov 8;11(1):2305378. doi: 10.1002/advs.202305378 (PMC10767407; doi:10.1002/advs.202305378)

checkCIF/PLATON report

You have not supplied any structure factors. As a result the full set of tests cannot be run.

THIS REPORT IS FOR GUIDANCE ONLY. IF USED AS PART OF A REVIEW PROCEDURE FOR PUBLICATION, IT SHOULD NOT REPLACE THE EXPERTISE OF AN EXPERIENCED CRYSTALLOGRAPHIC REFEREE.

No syntax errors found.   CIF dictionary   Interpreting this report

Datablock: 102

```
Bond precision:   C-C = 0.0142 A          Wavelength=0.71073

Cell:             a=31.7400(14)     b=25.0234(10)      c=26.3104(11)
                  alpha=90          beta=91.752(2)     gamma=90
Temperature:      273 K
```

	Calculated	Reported
Volume	20887.1(15)	20887.1(15)
Space group	C 2/c	C 1 2/c 1
Hall group	-C 2yc	-C 2yc
Moiety formula	C177 H123 N33 O38 Th6, 8(H2 O) [+ solvent]	C177 H119 N33 O36 Th6, 10(H2 O)
Sum formula	C177 H139 N33 O46 Th6 [+ solvent]	C177 H139 N33 O46 Th6
Mr	4856.46	4856.44
Dx,g cm-3	1.544	1.544
Z	4	4
Mu (mm-1)	4.333	4.333
F000	9360.0	9360.0
F000’	9191.79	
h,k,lmax	37,29,31	37,29,31
Nref	18393	18384
Tmin,Tmax		0.443,0.891
Tmin’		

```
Correction method= # Reported T Limits: Tmin=0.443 Tmax=0.891
AbsCorr = MULTI-SCAN

Data completeness= 1.000            Theta(max)= 25.000
```

R(reflections)= 0.0440(12288)   wR2(reflections)= 0.1220(18384)

S = 1.004   Npar= 1351

The following ALERTS were generated. Each ALERT has the format
test-name_ALERT_alert-type_alert-level.
Click on the hyperlinks for more details of the test.

Alert level C

```
RINTA01_ALERT_3_C  The value of Rint is greater than 0.12
            Rint given   0.120
PLAT053_ALERT_1_C Minimum Crystal Dimension Missing (or Error) ...     Please Check
PLAT054_ALERT_1_C Medium  Crystal Dimension Missing (or Error) ...     Please Check
PLAT055_ALERT_1_C Maximum Crystal Dimension Missing (or Error) ...     Please Check
PLAT220_ALERT_2_C NonSolvent   Resd 1  C   Ueq(max)/Ueq(min) Range        4.5 Ratio
PLAT222_ALERT_3_C NonSolvent Resd 1  H   Uiso(max)/Uiso(min) Range        4.3 Ratio
PLAT234_ALERT_4_C Large Hirshfeld Difference C23      --C31      .       0.19 Ang.
PLAT234_ALERT_4_C Large Hirshfeld Difference C31      --C49      .       0.18 Ang.
PLAT234_ALERT_4_C Large Hirshfeld Difference C66      --C75      .       0.18 Ang.
PLAT234_ALERT_4_C Large Hirshfeld Difference C88      --C89      .       0.21 Ang.
PLAT234_ALERT_4_C Large Hirshfeld Difference C88      --C90      .       0.20 Ang.
PLAT241_ALERT_2_C High   'MainMol' Ueq as Compared to Neighbors of        O12 Check
PLAT241_ALERT_2_C High   'MainMol' Ueq as Compared to Neighbors of        O15 Check
PLAT241_ALERT_2_C High   'MainMol' Ueq as Compared to Neighbors of        O18 Check
PLAT241_ALERT_2_C High   'MainMol' Ueq as Compared to Neighbors of        C63 Check
PLAT241_ALERT_2_C High   'MainMol' Ueq as Compared to Neighbors of        C68 Check
PLAT241_ALERT_2_C High   'MainMol' Ueq as Compared to Neighbors of        C88 Check
PLAT242_ALERT_2_C Low    'MainMol' Ueq as Compared to Neighbors of        C33 Check
PLAT242_ALERT_2_C Low    'MainMol' Ueq as Compared to Neighbors of        C82 Check
PLAT260_ALERT_2_C Large Average Ueq of Residue Including      O22      0.102 Check
PLAT260_ALERT_2_C Large Average Ueq of Residue Including      O23      0.121 Check
PLAT342_ALERT_3_C Low Bond Precision on  C-C Bonds ..............     0.0142 Ang.
PLAT369_ALERT_2_C Long   C(sp2)-C(sp2) Bond  C3       - C37      .       1.53 Ang.
```

Alert level G

```
PLAT002_ALERT_2_G Number of Distance or Angle Restraints on AtSite          3 Note
PLAT003_ALERT_2_G Number of Uiso or Uij Restrained non-H Atoms ...         37 Report
PLAT007_ALERT_5_G Number of Unrefined Donor-H Atoms .............          14 Report
PLAT020_ALERT_3_G The Value of Rint is Greater Than 0.12 .........      0.120 Report
PLAT042_ALERT_1_G Calc. and Reported MoietyFormula Strings  Differ     Please Check
PLAT176_ALERT_4_G The CIF-Embedded .res File Contains SADI Records          1 Report
PLAT186_ALERT_4_G The CIF-Embedded .res File Contains ISOR Records          2 Report
PLAT187_ALERT_4_G The CIF-Embedded .res File Contains RIGU Records          3 Report
PLAT199_ALERT_1_G Reported _cell_measurement_temperature ..... (K)        273 Check
PLAT200_ALERT_1_G Reported   _diffrn_ambient_temperature ..... (K)        273 Check
PLAT300_ALERT_4_G Atom Site Occupancy of O1         Constrained at        0.5 Check
PLAT300_ALERT_4_G Atom Site Occupancy of O1A        Constrained at        0.5 Check
PLAT300_ALERT_4_G Atom Site Occupancy of O5         Constrained at        0.5 Check
PLAT300_ALERT_4_G Atom Site Occupancy of O5A        Constrained at        0.5 Check
PLAT300_ALERT_4_G Atom Site Occupancy of O16        Constrained at        0.5 Check
PLAT300_ALERT_4_G Atom Site Occupancy of O16A       Constrained at        0.5 Check
PLAT300_ALERT_4_G Atom Site Occupancy of O19        Constrained at        0.5 Check
PLAT300_ALERT_4_G Atom Site Occupancy of O19A       Constrained at        0.5 Check
```

```
PLAT301_ALERT_3_G Main Residue  Disorder .............(Resd  1  )        17% Note
PLAT606_ALERT_4_G Solvent Accessible VOID(S) in Structure .......          ! Info
PLAT860_ALERT_3_G Number of Least-Squares Restraints ............        550 Note
PLAT869_ALERT_4_G ALERTS Related to the Use of SQUEEZE  Suppressed         ! Info
PLAT967_ALERT_5_G Note: Two-Theta Cutoff Value in Embedded .res ..      50.0 Degree
```

---

 0 **ALERT level A** = Most likely a serious problem - resolve or explain
 0 **ALERT level B** = A potentially serious problem, consider carefully
23 **ALERT level C** = Check. Ensure it is not caused by an omission or oversight
23 **ALERT level G** = General information/check it is not something unexpected

 6 ALERT type 1 CIF construction/syntax error, inconsistent or missing data
14 ALERT type 2 Indicator that the structure model may be wrong or deficient
 6 ALERT type 3 Indicator that the structure quality may be low
18 ALERT type 4 Improvement, methodology, query or suggestion
 2 ALERT type 5 Informative message, check

Datablock: 103

Bond precision: C-C = 0.0214 A   Wavelength=0.71073

Cell:	a=15.1500(5)	b=23.3651(8)	c=32.4213(8)
	alpha=90	beta=90	gamma=90
Temperature:	297 K		

	Calculated	Reported
Volume	11476.5(6)	11476.5(6)
Space group	P c c n	P c c n
Hall group	-P 2ab 2ac	-P 2ab 2ac
Moiety formula	C94 H72 N14 O37 Th6, 2(C H2 O2), 7(H2 O)	C94 H74 N14 O38 Th6, 6(H2 O), 2(C H2 O2)
Sum formula	C96 H90 N14 O48 Th6	C96 H90 N14 O48 Th6
Mr	3600.07	3600.05
Dx,g cm-3	2.084	2.084
Z	4	4
Mu (mm-1)	7.842	7.842
F000	6752.0	6752.0
F000’	6584.00	
h,k,lmax	18,27,38	18,27,38
Nref	10109	10094
Tmin,Tmax		0.026,0.055
Tmin’		

Correction method= # Reported T Limits: Tmin=0.026 Tmax=0.055
AbsCorr = MULTI-SCAN

Data completeness= 0.999 Theta(max)= 24.998

R(reflections)= 0.0556(7332) wR2(reflections)= 0.1596(10094)

S = 1.127 Npar= 760

The following ALERTS were generated. Each ALERT has the format
test-name_ALERT_alert-type_alert-level.
Click on the hyperlinks for more details of the test.

Alert level B

PLAT342_ALERT_3_B Low Bond Precision on C-C Bonds .... 0.02143 Ang.

Alert level C

```
PLAT042_ALERT_1_C Calc. and Reported MoietyFormula Strings  Differ     Please Check
PLAT220_ALERT_2_C NonSolvent   Resd 1   C    Ueq(max)/Ueq(min) Range      4.0 Ratio
PLAT220_ALERT_2_C NonSolvent   Resd 1   O    Ueq(max)/Ueq(min) Range      5.3 Ratio
PLAT232_ALERT_2_C Hirshfeld Test Diff (M-X)  Th3       --O9        .      5.2 s.u.
PLAT234_ALERT_4_C Large Hirshfeld Difference Th2       --O19       .     0.16 Ang.
PLAT234_ALERT_4_C Large Hirshfeld Difference O5        --C33       .     0.16 Ang.
PLAT241_ALERT_2_C High   'MainMol' Ueq as Compared to Neighbors of       O1 Check
PLAT241_ALERT_2_C High   'MainMol' Ueq as Compared to Neighbors of       O4 Check
PLAT241_ALERT_2_C High   'MainMol' Ueq as Compared to Neighbors of       O7 Check
PLAT241_ALERT_2_C High   'MainMol' Ueq as Compared to Neighbors of       O8 Check
PLAT241_ALERT_2_C High   'MainMol' Ueq as Compared to Neighbors of       O9 Check
PLAT241_ALERT_2_C High   'MainMol' Ueq as Compared to Neighbors of      C10 Check
PLAT241_ALERT_2_C High   'MainMol' Ueq as Compared to Neighbors of      C36 Check
PLAT241_ALERT_2_C High   'MainMol' Ueq as Compared to Neighbors of      C40 Check
PLAT242_ALERT_2_C Low    'MainMol' Ueq as Compared to Neighbors of      Th2 Check
PLAT242_ALERT_2_C Low    'MainMol' Ueq as Compared to Neighbors of      Th3 Check
PLAT242_ALERT_2_C Low    'MainMol' Ueq as Compared to Neighbors of      C46 Check
PLAT260_ALERT_2_C Large Average Ueq of Residue Including       O26    0.194 Check
PLAT260_ALERT_2_C Large Average Ueq of Residue Including       O18    0.134 Check
PLAT260_ALERT_2_C Large Average Ueq of Residue Including       O21    0.143 Check
PLAT260_ALERT_2_C Large Average Ueq of Residue Including       O25    0.110 Check
PLAT309_ALERT_2_C Single Bonded Oxygen (C-O > 1.3 Ang) ..........      O27 Check
PLAT314_ALERT_2_C Small Angle for H2O: Metal-O19      -H19A       .    92.17 Degree
```

Alert level G

```
PLAT002_ALERT_2_G Number of Distance or Angle Restraints on AtSite          6 Note
PLAT003_ALERT_2_G Number of Uiso or Uij Restrained non-H Atoms ...         12 Report
PLAT007_ALERT_5_G Number of Unrefined Donor-H Atoms ..............         17 Report
PLAT083_ALERT_2_G SHELXL Second Parameter in WGHT  Unusually Large     118.59 Why ?
PLAT172_ALERT_4_G The CIF-Embedded .res File Contains DFIX Records          2 Report
PLAT177_ALERT_4_G The CIF-Embedded .res File Contains DELU Records          5 Report
PLAT186_ALERT_4_G The CIF-Embedded .res File Contains ISOR Records          4 Report
PLAT187_ALERT_4_G The CIF-Embedded .res File Contains RIGU Records          2 Report
PLAT192_ALERT_3_G A Non-default DELU Restraint Value for First Par     0.0010 Report
PLAT192_ALERT_3_G A Non-default DELU Restraint Value for SecondPar     0.0010 Report
PLAT192_ALERT_3_G A Non-default DELU Restraint Value for First Par     0.0010 Report
PLAT192_ALERT_3_G A Non-default DELU Restraint Value for SecondPar     0.0010 Report
```

```
PLAT192_ALERT_3_G A Non-default DELU Restraint Value for First Par     0.0010 Report
PLAT192_ALERT_3_G A Non-default DELU Restraint Value for SecondPar     0.0010 Report
PLAT192_ALERT_3_G A Non-default DELU Restraint Value for First Par     0.0010 Report
PLAT192_ALERT_3_G A Non-default DELU Restraint Value for SecondPar     0.0010 Report
PLAT192_ALERT_3_G A Non-default DELU Restraint Value for First Par     0.0010 Report
PLAT192_ALERT_3_G A Non-default DELU Restraint Value for SecondPar     0.0010 Report
PLAT300_ALERT_4_G Atom Site Occupancy of O20       Constrained at        0.5 Check
PLAT300_ALERT_4_G Atom Site Occupancy of H20A      Constrained at        0.5 Check
PLAT300_ALERT_4_G Atom Site Occupancy of H20B      Constrained at        0.5 Check
PLAT300_ALERT_4_G Atom Site Occupancy of O21       Constrained at        0.5 Check
PLAT300_ALERT_4_G Atom Site Occupancy of H21A      Constrained at        0.5 Check
PLAT300_ALERT_4_G Atom Site Occupancy of H21B      Constrained at        0.5 Check
PLAT300_ALERT_4_G Atom Site Occupancy of O22       Constrained at        0.5 Check
PLAT300_ALERT_4_G Atom Site Occupancy of H22A      Constrained at        0.5 Check
PLAT300_ALERT_4_G Atom Site Occupancy of H22B      Constrained at        0.5 Check
PLAT300_ALERT_4_G Atom Site Occupancy of O24       Constrained at        0.5 Check
PLAT300_ALERT_4_G Atom Site Occupancy of H24A      Constrained at        0.5 Check
PLAT300_ALERT_4_G Atom Site Occupancy of H24B      Constrained at        0.5 Check
PLAT302_ALERT_4_G Anion/Solvent/Minor-Residue Disorder (Resd  4  )       100% Note
PLAT302_ALERT_4_G Anion/Solvent/Minor-Residue Disorder (Resd  5  )       100% Note
PLAT302_ALERT_4_G Anion/Solvent/Minor-Residue Disorder (Resd  6  )       100% Note
PLAT302_ALERT_4_G Anion/Solvent/Minor-Residue Disorder (Resd  7  )       100% Note
PLAT304_ALERT_4_G Non-Integer Number of Atoms in ..... (Resd  4  )       1.50 Check
PLAT304_ALERT_4_G Non-Integer Number of Atoms in ..... (Resd  5  )       1.50 Check
PLAT304_ALERT_4_G Non-Integer Number of Atoms in ..... (Resd  6  )       1.50 Check
PLAT304_ALERT_4_G Non-Integer Number of Atoms in ..... (Resd  7  )       1.50 Check
PLAT794_ALERT_5_G Tentative Bond Valency for Th1       (IV)      .       3.98 Info
PLAT794_ALERT_5_G Tentative Bond Valency for Th2       (IV)      .       4.37 Info
PLAT794_ALERT_5_G Tentative Bond Valency for Th3       (IV)      .       4.11 Info
PLAT794_ALERT_5_G Tentative Bond Valency for Th4       (IV)      .       3.94 Info
PLAT860_ALERT_3_G Number of Least-Squares Restraints ............         119 Note
PLAT933_ALERT_2_G Number of HKL-OMIT Records in Embedded .res File          7 Note
PLAT967_ALERT_5_G Note: Two-Theta Cutoff Value in Embedded .res ..       50.0 Degree
```

---

 0 **ALERT level A** = Most likely a serious problem - resolve or explain
 1 **ALERT level B** = A potentially serious problem, consider carefully
23 **ALERT level C** = Check. Ensure it is not caused by an omission or oversight
45 **ALERT level G** = General information/check it is not something unexpected

 1 ALERT type 1 CIF construction/syntax error, inconsistent or missing data
24 ALERT type 2 Indicator that the structure model may be wrong or deficient
12 ALERT type 3 Indicator that the structure quality may be low
26 ALERT type 4 Improvement, methodology, query or suggestion
 6 ALERT type 5 Informative message, check

Datablock: 104

Bond precision:	C-C = 0.0159 A		Wavelength=0.71073
Cell:	a=17.191(1)	b=28.528(1)	c=14.7593(8)
	alpha=90	beta=114.984(7)	gamma=90
Temperature:	297 K		

	Calculated	Reported
Volume	6561.0(7)	6561.0(7)
Space group	C 2/m	C 1 2/m 1
Hall group	-C 2y	-C 2y
Moiety formula	C56.58 H43.29 N7.66 O17.50 Th3, 0.658(C3 H7 N O), H2 O	C113.173 H80.587 N15.317 O32 Th6, 5(H2 O), 1.317(C3 H7 N O)
Sum formula	C58.55 H49.90 N8.32 O19.16 Th3	C117.12 H99.81 N16.64 O38.31 Th6
Mr	1872.68	3745.61
Dx,g cm-3	1.896	1.896
Z	4	2
Mu (mm-1)	6.858	6.858
F000	3530.8	3531.0
F000'	3446.62	
h,k,lmax	20,33,17	20,33,17
Nref	5915	5899
Tmin,Tmax		0.755,1.000
Tmin'		

Correction method= # Reported T Limits: Tmin=0.755 Tmax=1.000
AbsCorr = MULTI-SCAN

Data completeness= 0.997 Theta(max)= 25.000

R(reflections)= 0.0372(4882) wR2(reflections)= 0.1007(5899)

S = 1.032 Npar= 649

The following ALERTS were generated. Each ALERT has the format
test-name_ALERT_alert-type_alert-level.
Click on the hyperlinks for more details of the test.

Alert level C

```
PLAT041_ALERT_1_C Calc. and Reported SumFormula    Strings  Differ     Please Check
PLAT042_ALERT_1_C Calc. and Reported MoietyFormula Strings  Differ     Please Check
PLAT053_ALERT_1_C Minimum Crystal Dimension Missing (or Error) ...     Please Check
PLAT054_ALERT_1_C Medium  Crystal Dimension Missing (or Error) ...     Please Check
PLAT055_ALERT_1_C Maximum Crystal Dimension Missing (or Error) ...     Please Check
PLAT088_ALERT_3_C Poor Data / Parameter Ratio ...................        9.11 Note
PLAT213_ALERT_2_C Atom O2              has ADP max/min Ratio .....        3.6 prolat
PLAT220_ALERT_2_C NonSolvent   Resd 1  C   Ueq(max)/Ueq(min) Range        4.8 Ratio
PLAT220_ALERT_2_C NonSolvent   Resd 1  O   Ueq(max)/Ueq(min) Range        4.0 Ratio
PLAT222_ALERT_3_C NonSolvent Resd 1  H   Uiso(max)/Uiso(min) Range        4.8 Ratio
PLAT241_ALERT_2_C High   'MainMol' Ueq as Compared to Neighbors of         O2 Check
PLAT241_ALERT_2_C High   'MainMol' Ueq as Compared to Neighbors of         O6 Check
PLAT241_ALERT_2_C High   'MainMol' Ueq as Compared to Neighbors of        C23 Check
```

```
PLAT242_ALERT_2_C Low    'MainMol' Ueq as Compared to Neighbors of        Th3 Check
PLAT342_ALERT_3_C Low Bond Precision on  C-C Bonds ..............    0.01592 Ang.
```

Alert level G

```
FORMU01_ALERT_2_G  There is a discrepancy between the atom counts in the
            _chemical_formula_sum and the formula from the _atom_site* data.
            Atom count from _chemical_formula_sum:C117.12 H99.81 N16.64 O38.31 Th6
            Atom count from the _atom_site data:  C117.1080 H99.79202 N16.63199 O3
CELLZ01_ALERT_1_G Difference between formula and atom_site contents detected.
CELLZ01_ALERT_1_G ALERT: check formula stoichiometry or atom site occupancies.
           From the CIF: _cell_formula_units_Z    2
           From the CIF: _chemical_formula_sum  C117.12 H99.81 N16.64 O38.31 Th6
           TEST: Compare cell contents of formula and atom_site data

           atom    Z*formula  cif sites diff
           C        234.24    234.22    0.02
           H        199.62    199.58    0.04
           N         33.28     33.26    0.02
           O         76.62     76.63   -0.01
           Th        12.00     12.00    0.00
PLAT002_ALERT_2_G Number of Distance or Angle Restraints on AtSite          8 Note
PLAT003_ALERT_2_G Number of Uiso or Uij Restrained non-H Atoms ...         44 Report
PLAT004_ALERT_5_G Polymeric Structure Found with Maximum Dimension          1 Info
PLAT007_ALERT_5_G Number of Unrefined Donor-H Atoms .............           2 Report
PLAT045_ALERT_1_G Calculated and Reported Z Differ by a Factor ...          2 Check
PLAT068_ALERT_1_G Reported F000 Differs from Calcd (or Missing)...     Please Check
PLAT083_ALERT_2_G SHELXL Second Parameter in WGHT  Unusually Large      35.70 Why ?
PLAT172_ALERT_4_G The CIF-Embedded .res File Contains DFIX Records          5 Report
PLAT177_ALERT_4_G The CIF-Embedded .res File Contains DELU Records          4 Report
PLAT186_ALERT_4_G The CIF-Embedded .res File Contains ISOR Records          8 Report
PLAT187_ALERT_4_G The CIF-Embedded .res File Contains RIGU Records          1 Report
PLAT192_ALERT_3_G A Non-default DELU Restraint Value for First Par     0.0010 Report
PLAT192_ALERT_3_G A Non-default DELU Restraint Value for SecondPar     0.0010 Report
PLAT192_ALERT_3_G A Non-default DELU Restraint Value for First Par     0.0010 Report
PLAT192_ALERT_3_G A Non-default DELU Restraint Value for SecondPar     0.0010 Report
PLAT192_ALERT_3_G A Non-default DELU Restraint Value for First Par     0.0010 Report
PLAT192_ALERT_3_G A Non-default DELU Restraint Value for SecondPar     0.0010 Report
PLAT192_ALERT_3_G A Non-default DELU Restraint Value for First Par     0.0010 Report
PLAT192_ALERT_3_G A Non-default DELU Restraint Value for SecondPar     0.0010 Report
PLAT300_ALERT_4_G Atom Site Occupancy of O9         Constrained at        0.5 Check
PLAT300_ALERT_4_G Atom Site Occupancy of O9A        Constrained at        0.5 Check
PLAT300_ALERT_4_G Atom Site Occupancy of O10        Constrained at        0.5 Check
PLAT300_ALERT_4_G Atom Site Occupancy of O10A       Constrained at        0.5 Check
PLAT300_ALERT_4_G Atom Site Occupancy of O13        Constrained at        0.5 Check
PLAT300_ALERT_4_G Atom Site Occupancy of C2         Constrained at        0.5 Check
PLAT300_ALERT_4_G Atom Site Occupancy of C15        Constrained at        0.5 Check
PLAT300_ALERT_4_G Atom Site Occupancy of C26        Constrained at        0.5 Check
PLAT300_ALERT_4_G Atom Site Occupancy of C29        Constrained at        0.5 Check
PLAT300_ALERT_4_G Atom Site Occupancy of C30        Constrained at        0.5 Check
PLAT300_ALERT_4_G Atom Site Occupancy of H2         Constrained at        0.5 Check
PLAT300_ALERT_4_G Atom Site Occupancy of H15A       Constrained at        0.5 Check
PLAT300_ALERT_4_G Atom Site Occupancy of H15B       Constrained at        0.5 Check
PLAT300_ALERT_4_G Atom Site Occupancy of H15C       Constrained at        0.5 Check
PLAT300_ALERT_4_G Atom Site Occupancy of H26        Constrained at        0.5 Check
PLAT300_ALERT_4_G Atom Site Occupancy of H29        Constrained at        0.5 Check
PLAT300_ALERT_4_G Atom Site Occupancy of H30        Constrained at        0.5 Check
```

```
PLAT300_ALERT_4_G Atom Site Occupancy of H13B       Constrained at       0.25 Check
PLAT300_ALERT_4_G Atom Site Occupancy of H13C       Constrained at       0.25 Check
PLAT301_ALERT_3_G Main Residue  Disorder .............(Resd  1  )         46% Note
PLAT302_ALERT_4_G Anion/Solvent/Minor-Residue Disorder (Resd  2  )       100% Note
PLAT432_ALERT_2_G Short Inter X...Y Contact  O14      ..C9       .       2.99 Ang.
                                                 1-x,y,1-z  =      2_656 Check
PLAT432_ALERT_2_G Short Inter X...Y Contact  C23      ..C45      .       2.91 Ang.
                                                     x,y,z  =      1_555 Check
PLAT721_ALERT_1_G Bond    Calc     0.97000, Rep     0.95960 Dev...       0.01 Ang.
              C46      -H46C           1_555   1_555 ........     #  162 Check
PLAT789_ALERT_4_G Atoms with Negative _atom_site_disorder_group  #         44 Check
PLAT811_ALERT_5_G No ADDSYM Analysis: Too Many Excluded Atoms ....          ! Info
PLAT822_ALERT_4_G CIF-embedded .res Contains Negative PART Numbers          6 Check
PLAT860_ALERT_3_G Number of Least-Squares Restraints ............         328 Note
PLAT933_ALERT_2_G Number of HKL-OMIT Records in Embedded .res File          2 Note
PLAT941_ALERT_3_G Average HKL Measurement Multiplicity ..........         4.7 Low
PLAT967_ALERT_5_G Note: Two-Theta Cutoff Value in Embedded .res ..       50.0 Degree
```

---

0 **ALERT level A** = Most likely a serious problem - resolve or explain
0 **ALERT level B** = A potentially serious problem, consider carefully
15 **ALERT level C** = Check. Ensure it is not caused by an omission or oversight
53 **ALERT level G** = General information/check it is not something unexpected

10 ALERT type 1 CIF construction/syntax error, inconsistent or missing data
14 ALERT type 2 Indicator that the structure model may be wrong or deficient
14 ALERT type 3 Indicator that the structure quality may be low
26 ALERT type 4 Improvement, methodology, query or suggestion
4 ALERT type 5 Informative message, check

It is advisable to attempt to resolve as many as possible of the alerts in all categories. Often the minor alerts point to easily fixed oversights, errors and omissions in your CIF or refinement strategy, so attention to these fine details can be worthwhile. In order to resolve some of the more serious problems it may be necessary to carry out additional measurements or structure refinements. However, the purpose of your study may justify the reported deviations and the more serious of these should normally be commented upon in the discussion or experimental section of a paper or in the "special_details" fields of the CIF. checkCIF was carefully designed to identify outliers and unusual parameters, but every test has its limitations and alerts that are not important in a particular case may appear. Conversely, the absence of alerts does not guarantee there are no aspects of the results needing attention. It is up to the individual to critically assess their own results and, if necessary, seek expert advice.

Publication of your CIF in IUCr journals

A basic structural check has been run on your CIF. These basic checks will be run on all CIFs submitted for publication in IUCr journals (*Acta Crystallographica*, *Journal of Applied Crystallography*, *Journal of Synchrotron Radiation*); however, if you intend to submit to *Acta Crystallographica Section C* or *E* or *IUCrData*, you should make sure that full publication checks are run on the final version of your CIF prior to submission.

Publication of your CIF in other journals

Please refer to the *Notes for Authors* of the relevant journal for any special instructions relating to CIF submission.

PLATON version of 06/07/2023; check.def file version of 30/06/2023

Datablock 102 - ellipsoid plot

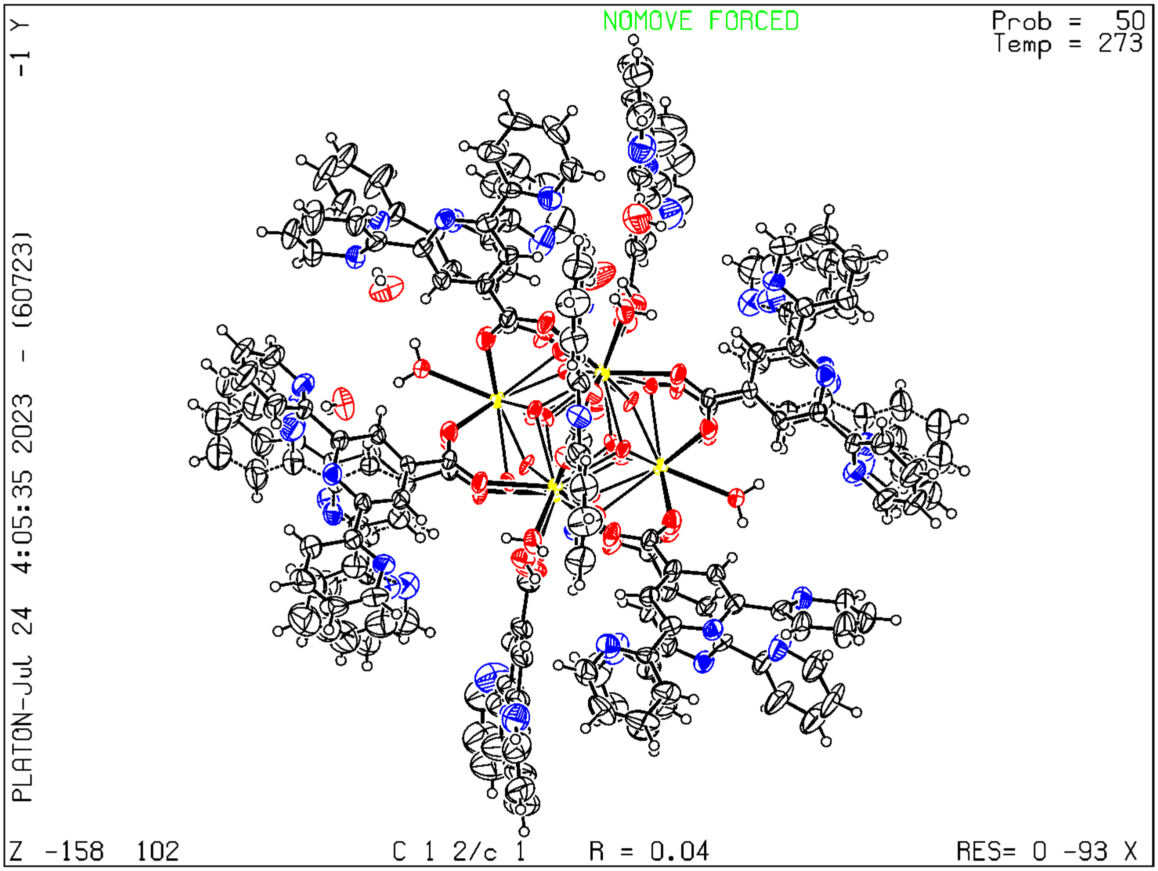

Datablock 103 - ellipsoid plot

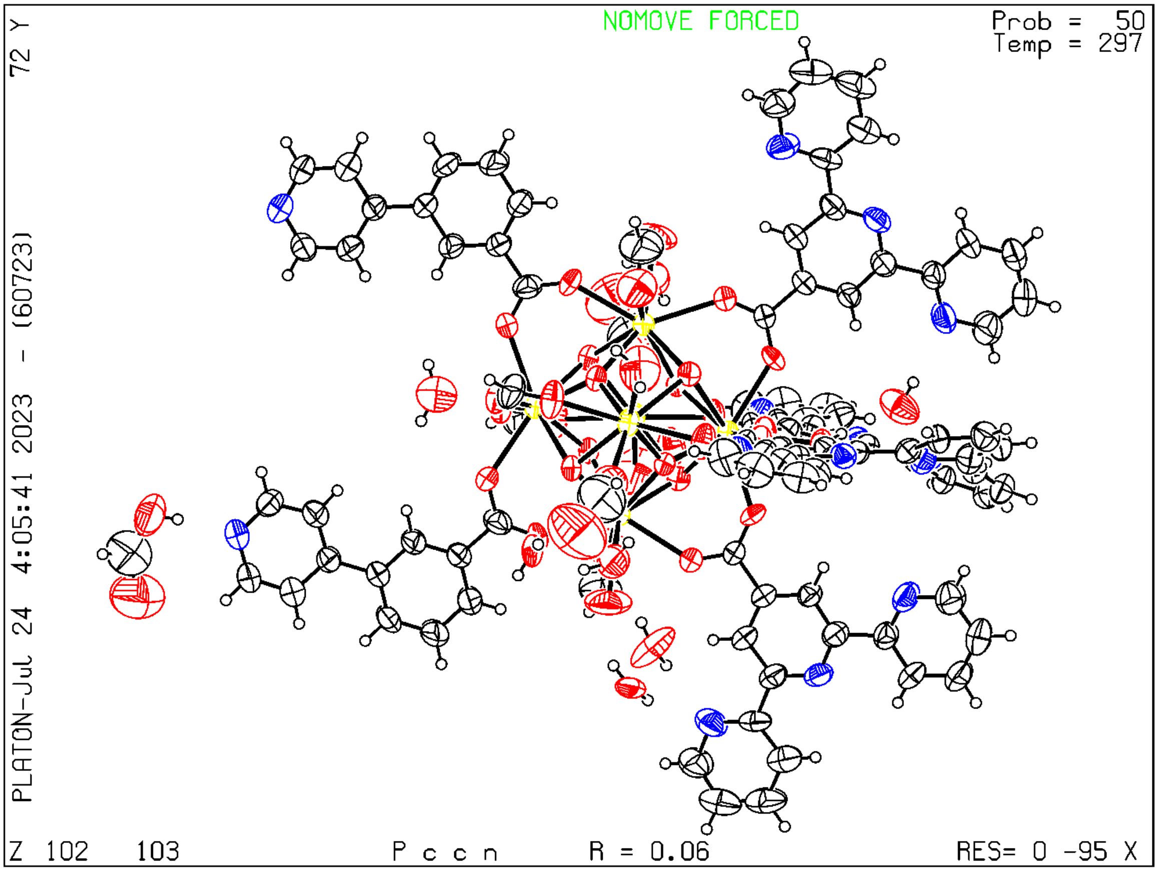

Datablock 104 - ellipsoid plot

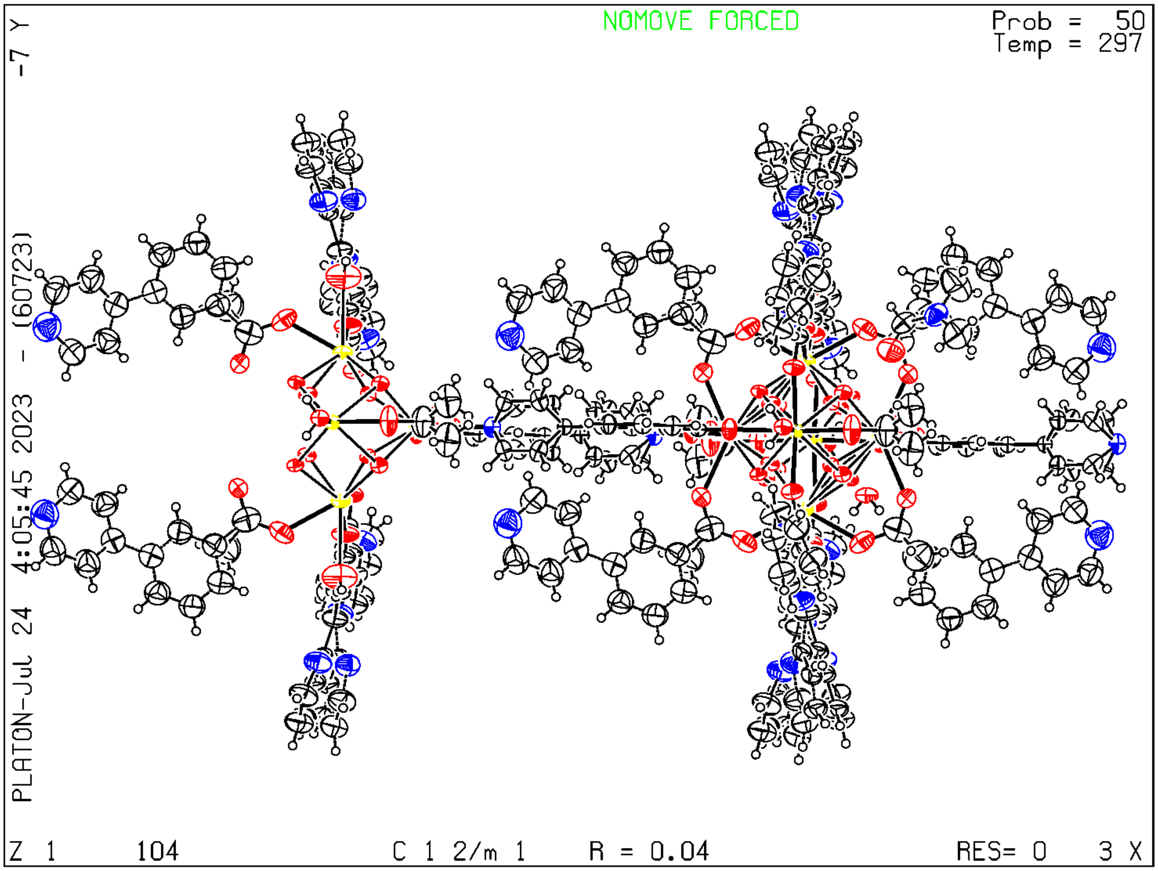

Supplement: Supplementary file 2 — Supporting Information [file ADVS-11-2305378-s001.zip › checkcif.pdf]
